# Tracking DNA methylation-based biological age over 8 years and its association with mortality in community-dwelling older adults

**DOI:** 10.1186/s13148-026-02067-3

**Published:** 2026-04-16

**Authors:** Qiming Yin, Ben Schöttker, Bernd Holleczek, Ziwen Fan, Joshua Stevenson-Hoare, Hermann Brenner

**Affiliations:** 1 Division of Clinical Epidemiology and Aging Research, German Cancer Research Center (DKFZ), Im Neuenheimer Feld 581, 69120 Heidelberg, Germany; 2 Medical Faculty Heidelberg, Heidelberg University, Im Neuenheimer Feld 672, 69120 Heidelberg, Germany; 3 Network Aging Research, Heidelberg University, Bergheimer Straße 20, 69115 Heidelberg, Germany; 4 Saarland Cancer Registry, Neugeländstraße 9, 66119 Saarbrücken, Germany; 5 German Cancer Research Center (DKFZ), Im Neuenheimer Feld 280, 69120 Heidelberg, Germany

**Keywords:** Biological age, Epigenetic clock, Longitudinal study, Life-course perspective, Aging

## Abstract

**Background:**

Population aging presents major health, social, economic, and political challenges. Aging is characterized by functional decline and increased disease risk. Recent advances in DNA methylation (DNAm) analysis have enabled more accurate estimates of biological age (BA), with accelerated epigenetic aging linked to unhealthy aging and higher mortality risk.

**Methods:**

We estimated DNAm-based BA using two-wave longitudinal data from 894 participants aged 50–75 years at baseline in the German ESTHER cohort, with a mean follow-up duration of 8.1 years. Cross-sectional correlations between chronological age (CA) and BA estimates based on five established epigenetic clocks were assessed. Average BA trajectories were modeled using linear regression. Multivariable linear regression was applied to identify potential baseline determinants of BA, and Cox proportional hazards models and restricted cubic splines (RCS) analyses were used to evaluate associations between BA dynamics and all-cause mortality.

**Results:**

BAs were correlated with baseline characteristics, including CA and sex. Longitudinally, BA increased at a slower rate than CA, and changes in BA were only weakly correlated with baseline CA. Smoking, physical activity, and alcohol consumption were identified as major determinants of individual BA trajectories. Furthermore, the rate of change in BA was significantly associated with all-cause mortality, with up to a 28% increased risk per standard deviation increase in BA slope.

**Conclusions:**

Our findings demonstrate strong correlations between BA and CA and highlight the influence of lifestyle factors on BA trajectories and mortality risk in older adults. We also emphasize the presence of sex-specific patterns in BA trajectories, underscoring the need for stratified approaches in aging research.

**Graphical abstract:**

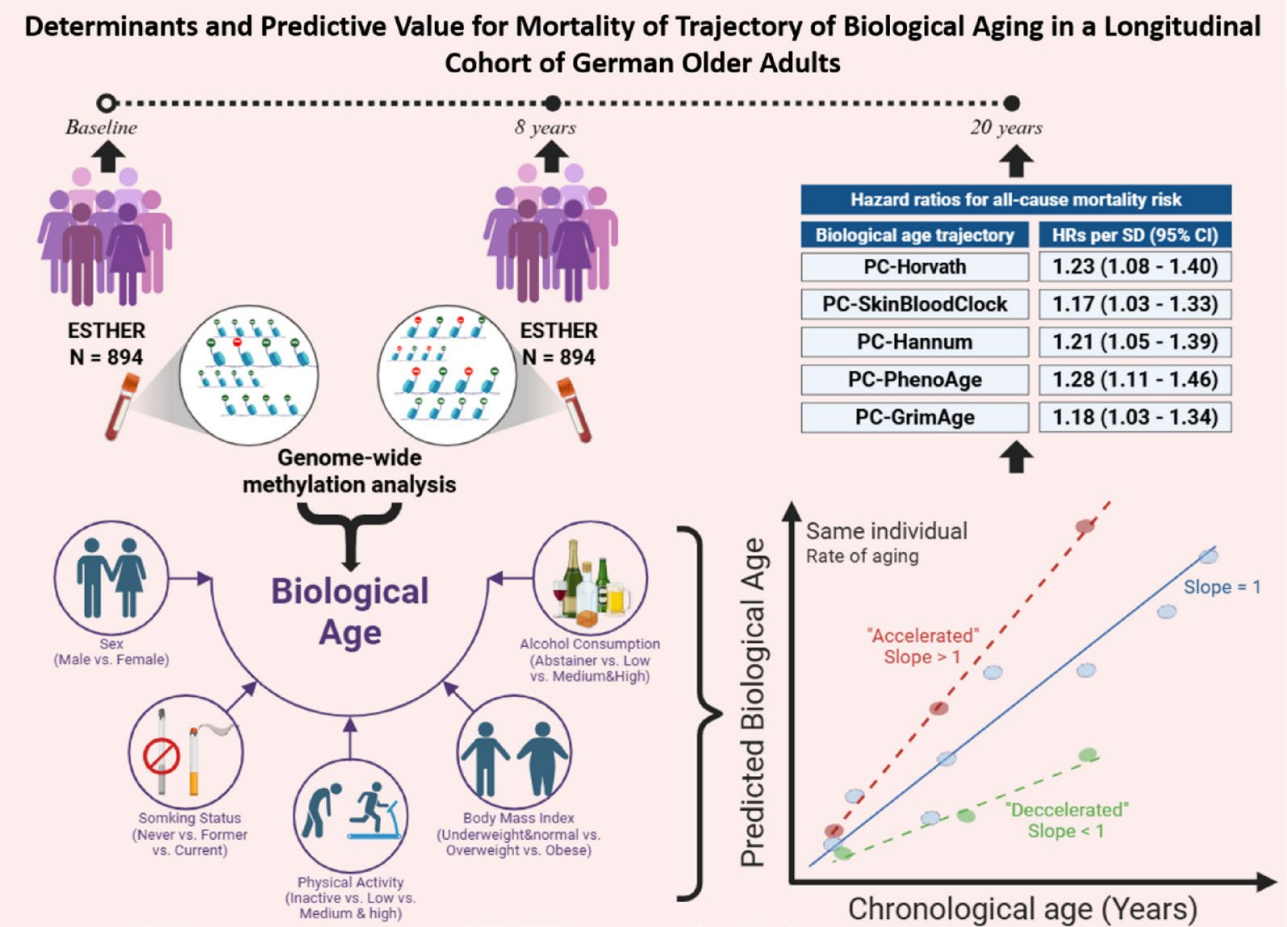

**Supplementary Information:**

The online version contains supplementary material available at 10.1186/s13148-026-02067-3.

## Background

Population aging is emerging as a major global health concern, contributing to complex challenges across medical, social, economic, and political domains. Aging is a gradual and continuous process marked by the progressive decline of physical and cognitive functions, along with an increased risk of major diseases. Advanced age is a well-established risk factor for cardiovascular, neurodegenerative, and malignant diseases [1].

Chronological age (CA) is an imperfect surrogate for biological aging, as individuals of the same age may exhibit substantial variation in functional status and disease risk [2–4]. Biological age (BA) has therefore been proposed as a more accurate indicator of an individual’s global physiological state, capturing variability in aging rates among individuals with the same CA. Therefore, BA can be used to assess health risks in individuals of the same age. BA can be estimated using DNA methylation (DNAm)-based epigenetic clocks, which are increasingly recognized as robust biomarkers of aging [2, 5, 6]. DNAm patterns change dynamically throughout the life course, driven by complex endogenous biological processes and influenced by external environmental exposures, such as aging, smoking, adiposity and lipid profiles, alcohol consumption, psychosocial factors and stressful environments [7]. These methylation signatures have been leveraged to quantify BA more precisely since they are likely capturing exposure- and time-specific information [7–9]. For a given CA, an advanced DNAm-based BA commonly referred to as epigenetic age acceleration - is typically associated with unhealthy aging, elevated mortality risk [10] and various age-related conditions such as frailty, and physical and cognitive disorder [11–18].

First-generation clocks, such as Hannum [8], Horvath [19], and SkinBloodClock [20] derive aging-related DNA methylation signals from specific cytosine-phosphate-guanine (CpG) dinucleotides. In contrast, second-generation clocks like PhenoAge and GrimAge incorporate additional information related to mortality risk and physiological biomarkers [21, 22]. However, many individual CpG sites measured on DNA methylation microarrays are subject to technical variability, which can compromise the reliability of epigenetic clock estimates [23]. To address this issue, principal components (PCs) derived from sets of CpGs, rather than individual CpGs, have been used to construct PC-based versions of epigenetic clocks (PC-clocks), which offer improved robustness, particularly in longitudinal analyses [24].

Nevertheless, most prior studies have relied on single time-point DNAm measurements, limiting our understanding of aging as a dynamic and time-dependent biological process. In the present study, we utilized two-wave longitudinal DNAm-based BA data, measured eight years apart, from a cohort of initially 50–75-year-old community-dwelling adults in Germany. Our primary aim was to identify baseline predictors of BA and to characterize trajectories of biological aging over time.

## Method

### Study population and data collection

Study participants were drawn from the ESTHER study, an ongoing population-based cohort study conducted in Saarland, Germany. Details of the study design have been described previously [25–27]. Briefly, 9940 individuals aged 50–75 years were recruited by their general practitioners (GPs) during routine health screenings between July 2000 and December 2002, and have been followed up every two to three years thereafter. At baseline and each follow-up, standardized self-administered questionnaires were used to collect information on sociodemographic characteristics, lifestyle, and dietary habits. General health examination results were documented by GPs using standardized forms. Blood samples were collected during examinations and stored at − 80 °C for future analyses. The ESTHER population has been shown to be representative of the general German population of the same age group (50–75 years) with respect to key sociodemographic variables and risk factor profiles [28]. For this study, a random sample of 900 participants with available blood samples at both baseline (T0) and 8-year follow-up (T1) was selected for epigenome-wide DNAm assessment. The ESTHER study was approved by the ethics committees of the Medical Faculty of the University of Heidelberg and the Medical Board of the State of Saarland. Written informed consent was obtained from all participants.

### Methylation assessment

Paired DNA samples were extracted from T0 and T1 blood samples using a salting-out procedure [29]. Genome-wide DNAm was assessed using the Infinium MethylationEPIC BeadChip Kit (Illumina, San Diego, CA, USA), according to the manufacturer’s instructions, by the Genomics and Proteomics Core Facility at the German Cancer Research Center (DKFZ), Heidelberg, Germany [17, 25, 30]. During preprocessing, probes targeting X or Y chromosomes were excluded and probes with detection *p*-value < 0.01 were defined missing value, and those participants with > 10% missing values were excluded [17, 25, 30]. After quality control and exclusion of low-quality samples, a total of 894 participants were retained for subsequent analyses.

### Calculation of DNAm aging algorithms

We computed five epigenetic age estimates using established DNA methylation clocks: Horvath Pan-Tissue, Horvath Skin & Blood, Hannum, PhenoAge, and GrimAge [8, 19–22]. Epigenetic age estimates and DNAm-based cell-type compositions were computed using the online DNAmAge calculator (https://dnamage.clockfoundation.org). To enhance longitudinal reliability, we used PC-based DNA methylation clocks as proposed by Higgins–Chen et al. [24]. In this approach, principal component analysis was applied to the CpG sites underlying each clock, and BA estimates were derived from the resulting components rather than from individual CpG-level measurements. This procedure reduces probe-specific technical noise and measurement error while preserving shared age-related biological signals. PC-based clocks have been shown to exhibit substantially greater stability across repeated measurements and were therefore particularly suitable for longitudinal analyses focusing on biological aging trajectories and rates of change. Missing CpG values (less than 5%) were imputed using mean substitution. Age acceleration (AgeAccel) was defined as the residual from regressing DNAm age estimates on CA [8].

### Longitudinal trajectory assessments

BA was measured longitudinally at two time points (T0 and T1). For each participant, the biological aging rate was estimated as the average annual change in DNA methylation age, calculated as the absolute difference in DNAm age (ΔDNAm age) between T0 and T1 divided by the individual time interval between measurements. This measure represents a linear approximation of the biological aging trajectory between the two time points and therefore implicitly assumes linear change over time. Given the availability of only two DNAm measurements, more complex nonlinear aging trajectories or short-term fluctuations could not be assessed.

### Mortality ascertainment

Vital status was ascertained via record linkage with population registries and local health authorities up to December 31, 2022. Follow-up for all-cause mortality was complete based on death certificates.

### Statistical methods

Baseline characteristics were summarized as means with standard deviations (SD) for continuous variables and proportions for categorical variables. Repeated measures correlation coefficients were calculated between CA and each of the five DNAm-based BAs, as well as their corresponding AgeAccels, and presented using a correlation matrix plot. Average BA trajectories were modeled using mixed-effects linear regression, with fixed effects for sex and CA, and a random intercept for each individual. Multivariable linear regression was used to examine baseline determinants of BA levels and BA slopes. Covariates included demographic characteristics, lifestyle factors, baseline BA, sex, education, and DNAm-estimated blood cell composition. Associations between BA slopes and all-cause mortality were estimated using Cox proportional hazards models with attained age as the time scale. The proportional hazards assumption was assessed using Schoenfeld residuals, and no substantial violations were observed. Left truncation and right censoring were accounted for, meaning individuals were only included if alive at the 8-year follow-up, which was used as the baseline for this analysis and they were followed-up until censoring at the date of death or at December 31, 2022. For comparability across clocks, BA slopes were standardized (mean = 0, SD = 1), allowing hazard ratios (HRs) to be interpreted per 1-SD increase in slope. Model 1 included univariate associations for each BA; Model 2 further adjusted for sex, education, smoking status, body mass index (BMI), physical activity, and alcohol consumption. To evaluate the exposure-response relationships between slope of BA and overall cancer incidence, restricted cubic spline (RCS) models with three knots at 25th, 50th and 75th percentiles were applied. We generated scatter plots to evaluate the relationship between the PC-clocks trajectories and the time to all-cause mortality. Because we examined the association of characteristics and all-cause mortality with the 5 PC-clocks, we used Bonferroni-correction to reduce the likelihood of a type 1 statistical error (false positive) according to the output of analyses. Analyses were conducted using the R software environment for statistical computing (version 4.2.0, Vienna, Austria).

## Results

### Study population and characteristics of BAs

A total of 894 participants from the ESTHER cohort were included in the analysis. These individuals had available DNAm data at both baseline (T0) and 8-year follow-up (T1), and their samples passed quality control. The average time interval between measurements was 8.1 years (range: 6.9–10.5 years) and a total of 299 deaths were observed during the subsequent follow-up period. Participant characteristics and PC-clocks at T0 and T1 are shown in Table 1. The mean ages were 61.2 years (SD = 6.3) at T0 and 69.2 years (SD = 6.3) at T1. Women accounted for 55.4% of the cohort, and 13.1% had received ≥ 12 years of school education. Over the 8-year period, the mean BMI did not change substantially (from 27.77 ± 4.56 at T0 to 27.99 ± 4.90 at T1). At baseline, 49.8% were never-smokers, and 38.6% reported medium or high levels of physical activity. The rate of increase in BA was slower than that of CA, as the changes in BA ranged from 4 to 4.9 years during the interval. Four of five clocks indicated older BA than CA at T0 (mean differences from 1.16 years for PCHorvath to around 10 years for PCGrimAge) and 2 at T1 (mean differences from 4.31 years for PCHannum to 7 years for PCGrimAge). PCPhenoAge was lower than CA at both T0 and T1, while PCGrimAge greater. The mean AgeAccel values were approximately zero, with SDs ranging from 3.75 (PCGrimAgeAccel at T0) to 7.10 (PCPhenoAgeAccel at T1) (Table 1).

**Table 1:** Descriptive statistics of participants at baseline and 8 years follow-up

	Baseline (2000–2002)						8 − year follow − up (2008–2010)					
	N	%	Mean	SD	Min	Max	N	%	Mean	SD	Min	Max
Chronological age	894		61.2	6.3	49	75			69.2	6.3	57	84
*Sex*												
Male	408	45.6						45.6				
Female	486	54.4						54.4				
*Nationality*												
German	840	94.0										
Non − German	54	6.0										
*Education* ^a^												
Low (≤ 9 years)	622	71.2										
Intermediate	137	15.7										
High (≥ 12 years)	114	13.1										
Height, (cm)	894		167.7	8.6	145	198						
Weight, (kg)	894		78.3	14.9	47.0	160	868		78.9	15.8	45.0	170
BMI, (kg/m^2^)	894		27.77	4.56	16.6	48.4	868		27.99	4.90	16.5	50.2
Smoking status^b^	866											
Never smoker	431	49.8										
Former smoker	320	37.0										
Current smoker	115	13.2										
Physical activities ^c^	890											
Inactive	151	17.0										
Low	395	44.4										
Medium or high	344	38.6										
Alcohol consumption^d^, (g/day)	823		11.0	14.4	0	163.9	788		9.55	12.92	0	98.9
*Leukocyte composition* ^e^												
CD8 + T cells	894		0.07	0.05	0	0.37			0.09	0.07	0	0.39
CD4 + T cells	894		0.18	0.08	0	0.50			0.21	0.11	0	0.65
NK cells	894		0.06	0.05	0	0.28			0.09	0.07	0	0.43
B cells	894		0.05	0.03	0	0.37			0.06	0.05	0	0.64
Monocytes	894		0.06	0.03	0	0.17			0.05	0.04	0	0.23
Granulocytes	894		0.64	0.11	0.28	1.00			0.55	0.16	0.02	1.03
*Biological age*												
PCHorvath	894		62.31	6.37	46.30	103.64			66.80	7.22	25.83	101.16
PCSkinBloodClock	894		64.33	6.44	48.48	103.91			68.34	7.66	27.94	100.59
PCHannum	894		68.72	6.58	51.27	101.35			73.51	7.74	27.00	105.13
PCPhenoAge	894		58.41	7.66	34.37	94.26			62.93	9.38	2.87	107.83
PCGrimAge	894		71.30	5.99	56.60	91.69			76.20	6.45	59.39	99.66
*Biological age acceleration*												
PCHorvathAgeAccel	894		0	4.39	− 10.46	41.44			0.01	5.31	− 38.39	34.56
PCSkinBloodClockAgeAccel	894		0	4.78	− 13.62	39.68			0.02	5.94	− 37.85	32.46
PCHannumAgeAccel	894		0	4.37	− 10.69	32.74			0.01	5.59	− 43.7	31.95
PCPhenoAgeAccel	894		0	5.34	− 15.96	35.98			0.03	7.10	56.82	45.29
PCGrimAgeAccel	894		− 0.01	3.75	− 8.41	20.49			0.01	3.96	− 14	23.77

BMI, body mass index; AgeAccel, age acceleration

^a^Data missing for 21 participants. ^b^Data missing for 28 participants. Never smoker was defined as an adult who has never smoked, or who has smoked less than 100 cigarettes in his or her lifetime; former smoker was defined as an adult who has smoked at least 100 cigarettes in his or her lifetime but who had quit smoking at the time of interview; current smoker was defined as an adult who has smoked 100 cigarettes in his or her lifetime and who currently smokes cigarettes. ^c^Data missing for 4 participants. Definition of inactive: < 1 h of physical activity/week, medium or high physical activity: ≥ 2 h of vigorous and ≥ 2 h of light physical activity/week, low physical activity: all other amounts of activity. ^d^Data missing for 71 participants. ^e^Estimated by the Houseman algorithm

### Correlations of CA and BAs

**Fig. 1:**
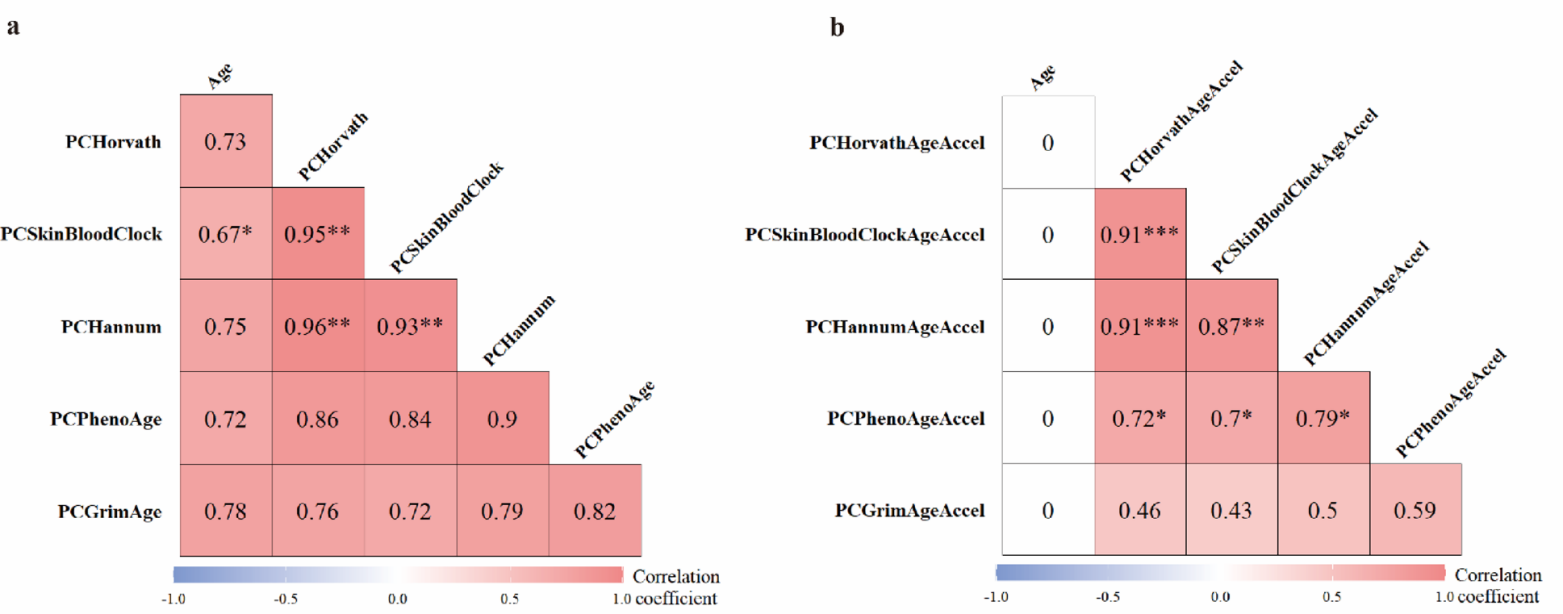
Heatmaps presenting correlation coefficients of BAs (**a**) and AgeAccels (**b**) with corresponding CA at T0. Red and blue tiles represent positive and negative correlations, respectively; color density indicates the magnitude of correlation coefficients. Statistical significance after Bonferroni-correction is indicated as follows: *p < 0.05, **p < 0.01, ***p < 0.001. AgeAccel, age acceleration

Correlation coefficients between BAs and CA were assessed at T0, presented in Fig. 1. In line with expectations, all BAs were moderately to strongly correlated with CA at T0 (*r* = 0.67–0.78, with PCSkinBloodClock showing the lowest correlations) and survive Bonferroni-correction (Fig. 1a). Strong inter-correlations were observed among BAs, with PCHorvath and PCHannum showing the highest mutual correlation (*r* = 0.96, *p* < 0.001 with Bonferroni correction), and PCGrimAge and PCSkinBloodClock the lowest (*r* = 0.72). As expected, no correlation for AgeAccels with CA while significant correlation between AgeAccels at baseline was observed as shown in Fig. 1b. Among AgeAccels, PCHorvathAgeAccel had the highest correlation with PCSkinBloodClockAgeAccel and PCHannumAgeAccel (*r* = 0.91, *p* < 0.001 with Bonferroni correction, Fig. 1b). Similar relationship of BAs and AgeAccels were also observed at T1, as presented in Additional File 1: Figure S1a and b.

Absolute change in BA between T0 and T1 was used to calculate ∆BAs, and BA slopes was calculated as ∆BA divided by interval length, which was interpreted as the average biologically age increase per year. Interestingly, higher biological aging rates were observed among older individuals, as there was a positive correlation of baseline CA with ∆BAs and BA slopes (*r* = 0.09–0.18, all *p*-values < 0.01 with four reaching the threshold of Bonferroni correction, Additional File 1: Figure S1 c and d). All ∆BAs and BA slopes were also significantly positively correlated with each other as expected.

### Longitudinal trajectories of BAs and ageaccels over 8 years

**Fig. 2:**
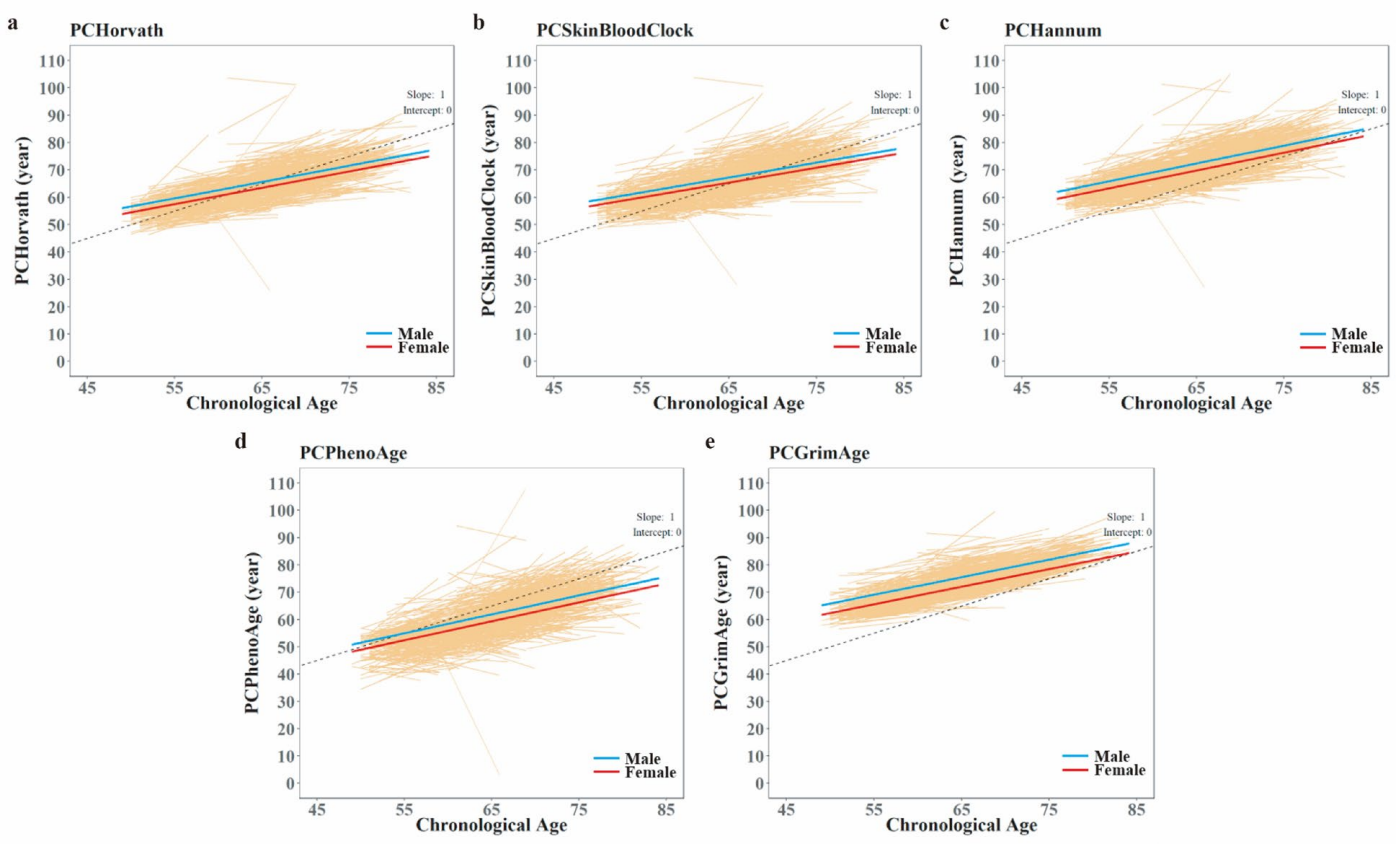
Longitudinal trajectories of individual and population level BA estimates. Individual BA estimates are presented as orange lines and sex-specific population BA as blue or red smooth line. Dashed line represents biologically aging if it was the same as chronological aging, with a slope of 1 and intercept of 0. IQR bands are included but are very narrow and so cannot be observed

Longitudinal changes in BA and AgeAccel were modeled using mixed-effects regression with random intercepts at the individual level. The use of PC-based clocks yielded smooth and consistent longitudinal aging trajectories across repeated measurements, supporting the robustness of the estimated biological aging rates. Figure 2 illustrates individual- and population-level BA trajectories across CA, stratified by sex. Although individual trends varied, population-level BA showed linear trajectories with slower pace compared with CA. As a result, PCHorvath and PCSkinBloodClock, which were higher than CA at T0, were younger than CA at the second measurement. Nevertheless, PCHannum and PCGrimAge were consistently higher, while PCPhenoAge younger than CA at both measurements (Table 1; Fig. 2). Sex differences were also evident: women consistently showed significantly lower BA values than men across all PC clocks (*p*-values ≤ 1.15E-7). However, the interaction term between CA and sex was not statistically significant, indicating similar trajectory shapes between sexes. The trajectories for AgeAccels were also independent from CA, and women also showed significantly lower values than men. There were no significant interaction effects between CA and gender across all PC AgeAccels (Additional File 1: Figure S2).

### Cross-sectional analyses: associations of life style factors with BAs at baseline and follow-up

Cross-sectional associations between lifestyle factors and BAs at T0 and T1 are presented in Tables 2 and 3. Intermediate and high education was generally associated with lower BAs at both measurements, whereas male sex, overweight and obesity, and smoking were generally associated with increased BAs.

**Table 2:** Associations of baseline characteristics with baseline PC-clocks

Variants	PCHorvath			PCSkinBloodClock		PCHannum		PCPhenoAge		PCGrimAge	
	β (95%CI)	*p-*value		β (95%CI)	*p*-value	β (95%CI)	*p-*value	β (95%CI)	*p-*value	β (95%CI)	*p-*value
*Sex*											
Female	ref	–		ref	–	ref	–	ref	–	ref	–
Male	1.22 (0.58–1.85)	**2.02E−4**		0.51 (− 0.14–1.16)	0.124	1.39 (0.78–2.01)	**1.05E−5**	0.88 (0.21–1.56)	**0.010**	2.34 (1.95–2.72)	**7.67E−30**
*Education*											
Low (≤ 9 years)	ref	–		ref	–	ref	–	ref	–	ref	–
Intermediate	− 0.47 (− 1.06–0.12)	0.115		− 0.36 (− 0.97–0.24)	0.236	− 0.60 (− 1.17– − 0.03)	0.038	− 0.47 (− 1.10–0.15)	0.134	− 0.57 (− 0.93– − 0.22)	**0.002**
High (≥ 12 years)	− 0.27 (− 0.92–0.39)	0.423		− 0.04 (− 0.71–0.63)	0.910	− 0.23 (− 0.86–0.40)	0.475	0.21 (− 0.48–0.90)	0.555	− 0.26 (− 0.66–0.14)	0.198
*BMI*											
Underweight & Normal range (< 25)	ref	–		ref	–	ref	–	ref	–	ref	–
Overweight (< 30)	0.14 (− 0.41–0.69)	0.605		0.18 (− 0.39–0.74)	0.541	0.29 (− 0.24–0.82)	0.280	0.84 (0.26–1.42)	**0.005**	0.52 (0.19–0.85)	**0.002**
Obese (≥ 30)	0.48 (0.02–0.93)	0.040		0.49 (0.03–0.96)	0.039	0.40 (− 0.04–0.84)	0.074	0.50 (0.02–0.98)	0.041	0.05 (− 0.22–0.33)	0.699
*Smoking status*											
Never smoker	ref	–		ref	–	ref	–	ref	–	ref	–
Former smoker	0.03 (− 0.61–0.66)	0.938		0.11 (− 0.54–0.76)	0.738	0.18 (− 0.44–0.79)	0.572	0.39 (− 0.28–1.06)	0.258	1.58 (1.19–1.97)	**3.77E−15**
Current smoker	1.10 (0.19–2.01)	0.018		1.52 (0.59–2.45)	**0.001**	1.28 (0.40–2.16)	**0.004**	2.28 (1.32–3.24)	**3.90E−6**	5.74 (5.19–6.29)	**1.94E−74**
*Physical activities*											
Inactive	ref	–		ref	–	ref	–	ref	–	ref	–
Low	− 0.60 (− 1.27–0.07)	0.080		− 0.70 (− 1.39– − 0.02)	0.044	− 0.58 (− 1.23–0.06)	0.077	− 1.07 (− 1.78– − 0.37)	**0.003**	− 0.46 (− 0.86– − 0.05)	0.028
Medium or high	0.34 (− 0.15–0.84)	0.175		0.43 (− 0.08–0.94)	0.099	0.27 (− 0.21–0.75)	0.269	0.55 (0.02–1.07)	0.042	0.23 (− 0.08–0.53)	0.143
*Alcohol consumption* ^a^											
Abstainer	ref	–		ref	–	ref	–	ref	–	ref	–
Low	0.20 (− 0.66–1.06)	0.646		0.03 (− 0.84–0.91)	0.939	0.23 (− 0.60–1.06)	0.586	0.38 (− 0.52–1.29)	0.409	− 0.02 (− 0.54–0.50)	0.945
Medium or High	− 0.02 (− 0.59–0.54)	0.933		− 0.10 (− 0.68–0.48)	0.733	0.21 (− 0.34–0.75)	0.458	0.47 (− 0.12–1.06)	0.121	0.14 (− 0.20–0.49)	0.410

β and *p*-value were estimated by multivariate regression adjusted for covariates.The bold *p*-value means pass Bonferroni-correction

BMI body mass index

^a^Data missing for 71 participants. The consumption of alcohol was calculated by the following equation: 1 bottle of beer = 11.88 g ethanol, 1 glass of wine = 22.0 g ethanol, 1 shot of liquor = 6.4 g ethanol. Abstainer was without any alcohol consumption. Women 0–19.99 g/day or men 0–39.99 g/day were low consumption, and women ≥ 20 g/day or men ≥ 40 g/day were medium or high consumption

**Table 3:** Associations of baseline characteristics with 8-year follow-up PC-clocks

Variants	PCHorvath		PCSkinBloodClock		PCHannum		PCPhenoAge		PCGrimAge	
	β (95%CI)	*p-*value	β (95%CI)	*p-*value	β (95%CI)	*p-*value	β (95%CI)	*p-value*	β (95%CI)	*p-*value
*Sex*										
Female	ref	−	ref	−	ref	−	ref	−	ref	−
Male	0.99 (0.24–1.75)	**0.010**	0.18 (− 0.60–0.96)	0.653	1.14 (0.39–1.89)	**0.003**	0.61 (− 0.20–1.42)	0.142	2.22 (1.83–2.60)	**6.79E−28**
*Education*										
Low (≤ 9 years)	ref	−	ref	−	ref	−	ref	−	ref	−
Intermediate	− 0.65 (− 1.34–0.03)	0.063	− 0.53 (− 1.24–0.19)	0.148	− 0.73 (− 1.41– − 0.04)	0.037	− 0.72 (− 1.46–0.02)	0.056	− 0.50 (− 0.85– − 0.15)	**0.005**
High (≥ 12 years)	− 0.53 (− 1.29–0.24)	0.175	− 0.35 (− 1.15–0.44)	0.381	− 0.53 (− 1.29–0.23)	0.172	− 0.21 (− 1.03–0.62)	0.621	− 0.25 (− 0.64–0.14)	0.204
*BMI*										
Underweight & Normal range (< 25)	ref	−	ref	−	ref	−	ref	−	ref	−
Overweight (< 30)	− 0.03 (− 0.67–0.61)	0.934	− 0.06 (− 0.72–0.61)	0.865	0.20 (− 0.43–0.84)	0.528	0.82 (0.13–1.51)	0.021	0.55 (0.23–0.88)	**8.89E−4**
Obese (≥ 30)	0.59 (0.06–1.12)	0.029	0.66 (0.11–1.21)	0.019	0.61 (0.08–1.14)	0.023	0.68 (0.10–1.25)	0.021	0.13 (− 0.14–0.40)	0.349
*Smoking status*										
Never smoker	ref	−	ref	−	ref	−	ref	−	ref	−
Former smoker	0.47 (− 0.27–1.22)	0.210	0.51 (− 0.26–1.28)	0.192	0.57 (− 0.17–1.31)	0.128	0.66 (− 0.14–1.46)	0.104	1.44 (1.06–1.81)	**1.64E−13**
Current smoker	2.36 (1.32–3.41)	**9.91E−6**	2.69 (1.61–3.78)	**1.26E−6**	2.47 (1.43–3.50)	**3.38E−6**	3.21 (2.09–4.34)	**2.83E−8**	5.21 (4.69–5.74)	**1.69E−68**
*Physical activities*										
Inactive	ref	−	ref	−	ref	−	ref	−	ref	−
Low	− 0.55 (− 1.33–0.23)	0.169	− 0.54 (− 1.36–0.27)	0.188	− 0.65 (− 1.43–0.12)	0.099	− 1.00 (− 1.84– − 0.16)	0.020	− 0.39 (− 0.79–0)	0.051
Medium or high	0.43 (− 0.15–1.01)	0.145	0.53 (− 0.07–1.14)	0.083	0.45 (− 0.13–1.02)	0.129	0.65 (0.02–1.27)	0.042	0.23 (− 0.07–0.52)	0.134
*Alcohol consumption* ^a^										
Abstainer	ref	−	ref	−	ref	−	ref	−	ref	−
Low	− 0.82 (− 1.81–0.18)	0.107	− 1.09 (− 2.13– − 0.06)	0.038	− 0.79 (− 1.78–0.20)	0.117	− 0.61 (− 1.68–0.47)	0.267	− 0.18 (− 0.69–0.32)	0.474
Medium or High	− 0.57 (− 1.23–0.08)	0.087	− 0.73 (− 1.41– − 0.05)	0.036	− 0.47 (− 1.12–0.18)	0.156	− 0.16 (− 0.87–0.55)	0.658	0.02 (− 0.32–0.35)	0.929

β and *p*-value were estimated by multivariate regression adjusted for covariates

BMI, body mass index

^a^Data missing for 71 participants. The consumption of alcohol was calculated by the following equation: 1 bottle of beer = 11.88 g ethanol, 1 glass of wine = 22.0 g ethanol, 1 shot of liquor = 6.4 g ethanol. Abstainer was without any alcohol consumption. Women 0–19.99 g/day or men 0–39.99 g/day were low consumption, and women ≥ 20 g/day or men ≥ 40 g/day were medium or high consumption

Furthermore, we performed subgroup analyses by sex since we observed sex interaction with BMI, smoking and physical activity. Subgroup analyses by sex revealed that smoking was significantly associated with higher BAs in both men and women. However, this smoking-related elevation in BA was observed across all five PC-clocks in men, whereas in smoking women, only an older PCGrimAge was observed at both measurements (*p* ≤ 1.08E-6, Additional File 1: Tables S1–S4). Intermediate education was significantly associated with lower PCGrimAge in men only (β < 0, with *p* ≤ 0.004, Additional File 1: Tables S1 and S3). In women at both time points, higher BMI was associated with significantly higher BAs compared with normal or underweight, while low level physical activity was associated with younger BA (*p* ≤ 0.010, Additional File 1: Tables S1-S4). Corresponding patterns were also observed for AgeAccel (Additional File 1: Tables S1–S10).

### Longitudinal analyses: BA slope and lifestyle determinants

To examine predictors of BA change over time, we performed multivariable regression analyses of BA slopes calculated via absolute changes in BA over interval and divided by individual interval. Education, BMI, and physical activity were not significantly associated with BA slopes, although the directions of association were consistent with those observed cross-sectionally (Table 4). Smoking and male sex were associated with steeper BA slopes (β > 0, *p*-values = 1.20E-4–0.041), although just current smoking reached the significance threshold after Bonferroni correction. Medium or high level of alcohol consumption showed a significant negative association with slopes compared to alcohol abstainers.

**Table 4:** Associations between baseline characteristics and age acceleration slope during follow-up interval among all participants

Variants	PCHorvath slope		PCSkinBloodClock slope		PCHannum slope		PCPhenoAge slope		PCGrimAge slope	
	β (95%CI)	*p-*value	β (95%CI)	*p-*value	β (95%CI)	*p-*value	β (95%CI)	*p-*value	β (95%CI)	*p-*value
*Sex*										
Female	ref	−	ref	−	ref	−	ref	−	ref	−
Male	0.06 (0–0.12)	0.041	0.07 (0.01–0.13)	0.035	0.08 (0.02–0.15)	0.015	0.11 (0.01–0.22)	0.038	0.02 (− 0.03–0.06)	0.441
*Education*										
Low (≤ 9 years)	ref	−	ref	−	ref	−	ref	−	ref	−
Intermediate	− 0.02 (− 0.08–0.03)	0.357	− 0.02 (− 0.08–0.04)	0.444	− 0.02 (− 0.09–0.04)	0.447	− 0.02 (− 0.12–0.07)	0.644	0.01 (− 0.03–0.05)	0.487
High (≥ 12 years)	− 0.04 (− 0.10–0.02)	0.161	− 0.03 (− 0.10–0.03)	0.322	− 0.05 (− 0.12–0.02)	0.172	− 0.07 (− 0.18–0.03)	0.178	− 0.02 (− 0.06–0.03)	0.512
*BMI*										
Underweight & Normal range (< 25)	ref	−	ref	−	ref	−	ref	−	ref	−
Overweight (< 30)	0.01 (− 0.04–0.06)	0.797	0.01 (− 0.05–0.06)	0.793	0.02 (− 0.04–0.08)	0.469	0.05 (− 0.04–0.14)	0.268	0.02 (− 0.01–0.06)	0.211
Obese (≥ 30)	0.01 (− 0.03–0.05)	0.517	0.02 (− 0.03–0.07)	0.406	0.03 (− 0.02–0.08)	0.248	0.03 (− 0.04–0.11)	0.419	0.02 (− 0.02–0.05)	0.338
*Smoking status*										
Never smoker	ref	−	ref	−	ref	−	ref	−	ref	−
Former smoker	0.06 (0–0.12)	0.038	0.07 (0.01–0.14)	0.026	0.06 (− 0.01–0.13)	0.078	0.08 (− 0.02–0.19)	0.119	0 (− 0.04–0.05)	0.837
Current smoker	0.16 (0.08–0.24)	**1.20E−4**	0.17 (0.08–0.26)	**3.38E−4**	0.17 (0.08–0.27)	**4.57E−4**	0.23 (0.08–0.38)	**0.002**	− 0.02 (− 0.08–0.04)	0.532
Physical activities										
Inactive	ref	−	ref	−	ref	−	ref	−	ref	−
Low	− 0.01 (− 0.07–0.05)	0.789	− 0.02 (− 0.09–0.05)	0.586	− 0.04 (− 0.11–0.03)	0.299	− 0.05 (− 0.16–0.06)	0.402	0 (− 0.05–0.05)	0.966
Medium or high	0.03 (− 0.02–0.07)	0.218	0.03 (− 0.02–0.08)	0.200	0.05 (0–0.10)	0.050	0.08 (0–0.17)	0.048	0.03 (− 0.01–0.06)	0.137
*Alcohol consumption* ^a^										
Abstainer	ref	−	ref	−	ref	−	ref	−	ref	−
Low	− 0.12 (− 0.20– − 0.04)	**0.002**	− 0.14 (− 0.23– − 0.05)	**0.001**	− 0.14 (− 0.23– − 0.05)	**0.002**	− 0.19 (− 0.33– − 0.05)	**0.009**	− 0.04 (− 0.10–0.02)	0.149
Medium or High	− 0.06 (− 0.11– − 0.01)	0.017	− 0.06 (− 0.12– − 0.01)	0.032	− 0.09 (− 0.14– − 0.03)	**0.005**	− 0.12 (− 0.21– − 0.03)	0.013	− 0.03 (− 0.07–0.01)	0.130

β and *p*-value were estimated by multivariate regression adjusted for covariates.The bold *p*-value means passed Bonferroni-correction

BMI, body mass index

^a^Data missing for 71 participants. The consumption of alcohol was calculated by the following equation: 1 bottle of beer = 11.88 g ethanol, 1 glass of wine = 22.0 g ethanol, 1 shot of liquor = 6.4 g ethanol. Abstainer was without any alcohol consumption. Women 0–19.99 g/day or men 0–39.99 g/day were low consumption, and women ≥ 20 g/day or men ≥ 40 g/day were medium or high consumption

**Fig. 3:**
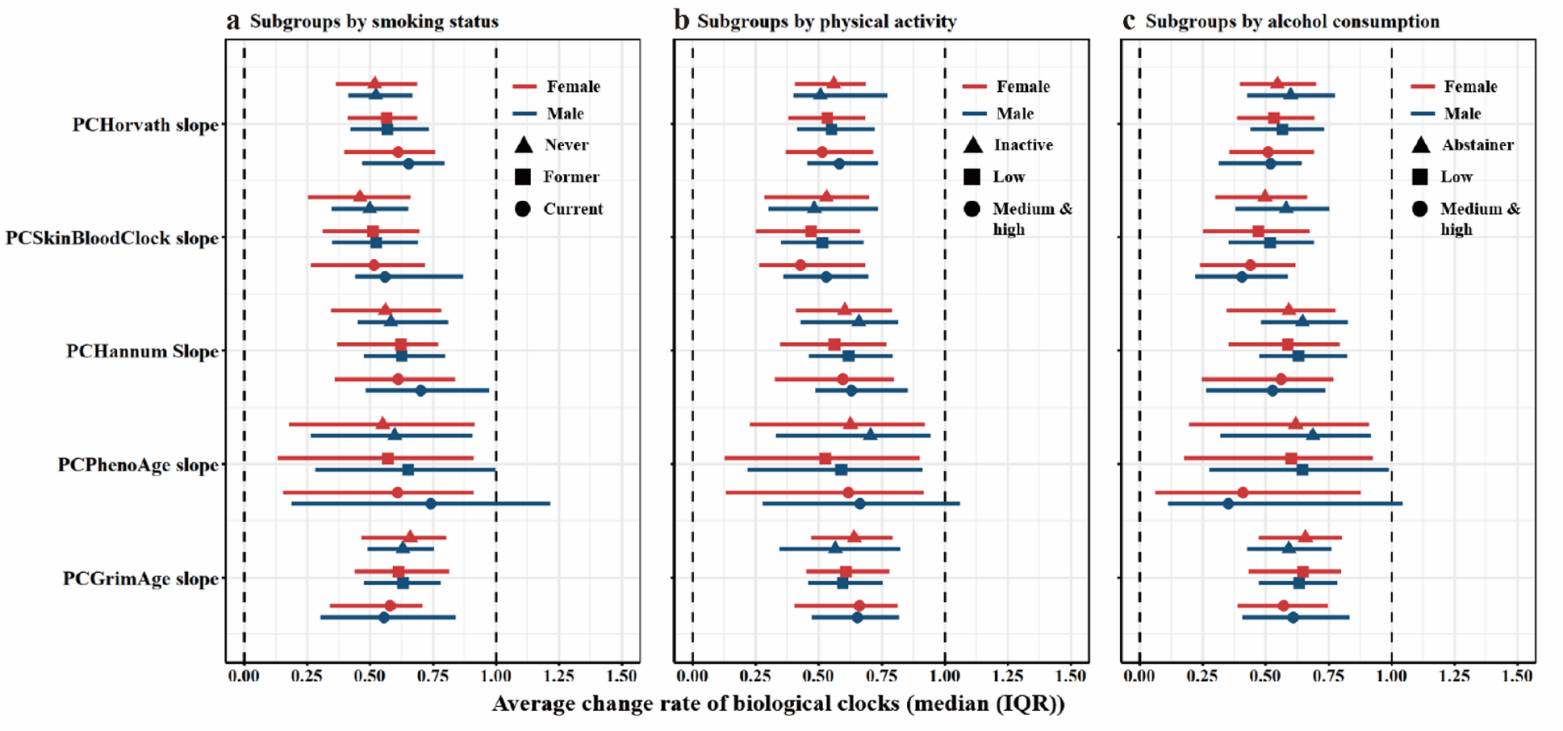
Distributions and survival analyses of BA slopes in subgroups. The forest plots present distributions of the average changing rate (slope) of BAs stratified by sex, baseline smoking status (**a**), physical activity (**b**) and alcohol consumption (**c**) The points and horizontal lines denoted median (IQR), the point shapes represented subgroups and colors represented sex

Subgroup analyses suggested a moderating effect of sex. Associations of both smoking and alcohol consumption with BA were generally stronger in men, except for PCGrimAge. Physical activity tended to be associated with a slower biological aging rate in women (β < 0) and a higher biological aging rate in men (β > 0), although *p*-values did not reach threshold after Bonferroni-correction in either gender (Fig. 3, Additional File 1: Tables S11–S12).

### Longitudinal analysis: BAs and all-cause mortality

**Fig. 4:**
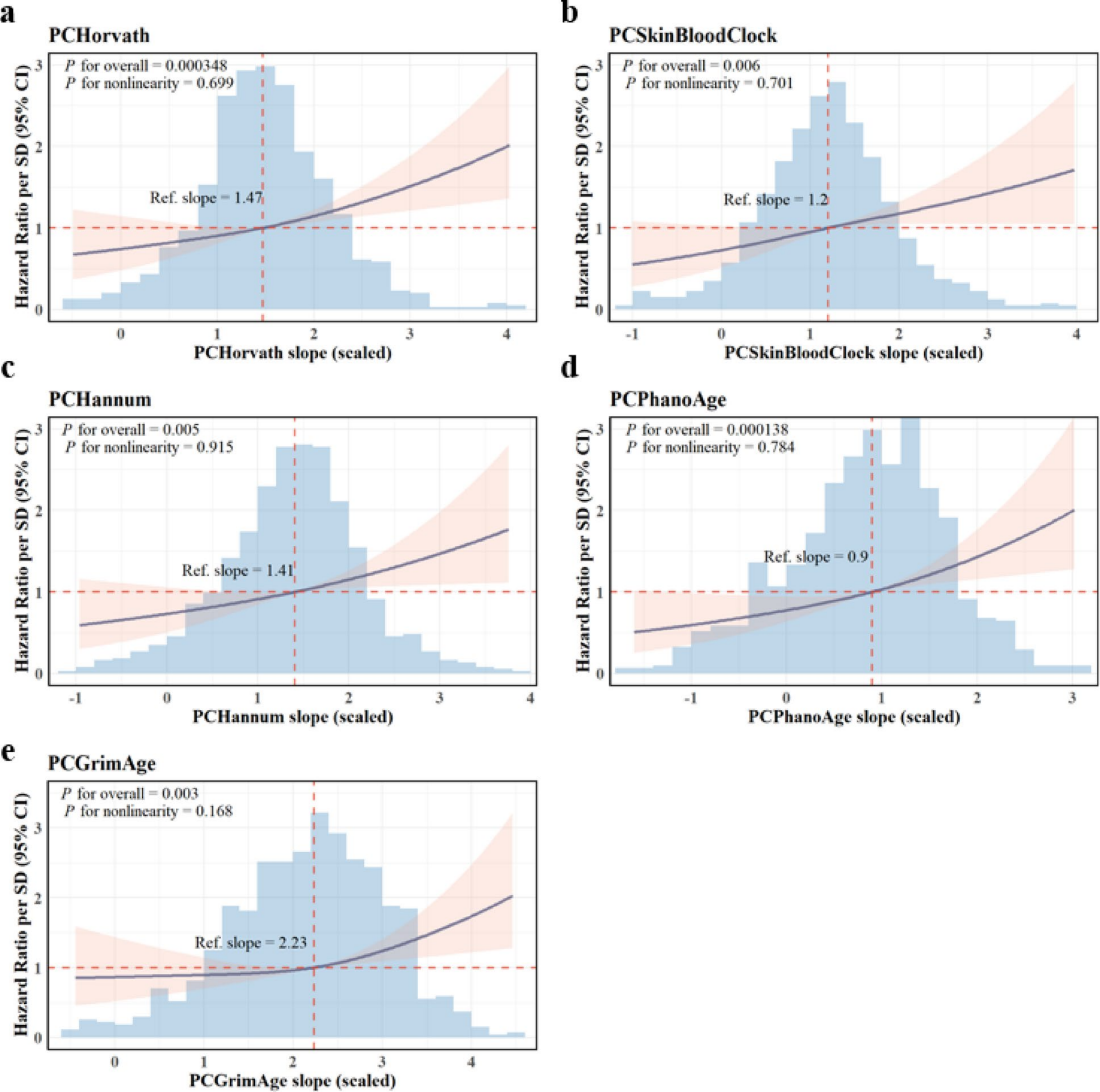
Dose-response relationships of PC-clock slopes with all-cause mortality risk. The models were adjusted for age, sex, nationality, leukocyte composition, education level, BMI, smoking status, alcohol consumption, BMI and physical activity

We further assessed the association of BA slopes and all-cause mortality using Cox proportional hazards models, with age as the time scale. BA slopes were standardized (mean = 0, SD = 1) to enable direct comparison of HRs, which reflected the mortality risk associated with a one-SD increase in BA slope. The median follow-up time was 8.1 years (range: 6.9–10.5 years) and a total of 299 deaths were observed during the follow-up period. Fully adjusted models included sex, education, smoking status, BMI, physical activity, and alcohol consumption. In both univariate and multivariate models, all five PC-based clocks showed significant associations with increased mortality in the total population. A one-SD increase in slope was associated with a 17–28% increased risk of death (Additional File 1: Table S13). Restricted cubic spline analysis demonstrated a trend of increasing mortality risk with steeper trajectory of biological aging (Fig. 4). The risk increase steeper at higher trajectories of biologically aging, the analysis did not provide significant evidence of significant nonlinearity (*p* for nonlinearity > 0.05) across all five epigenetic clocks (all p for overall < 0.01). Sex-specific survival analyses revealed generally stronger associations in men, in whom the slope of PCPhenoAge was significantly associated with higher all-cause mortality even after fully adjustment.

## Discussion

In this study, we comprehensively examined five PC-based DNAm biological clocks using two-wave longitudinal data from a cohort of community-dwelling older adults. Our findings demonstrate strong associations between DNAm-based BA clocks and CA, as well as key demographic and lifestyle factors, including sex, education, BMI, smoking, physical activity, and alcohol consumption. All five biological aging clocks (BAs) were highly correlated with CA at both time points, while ∆BA (changes over time) showed only modest associations with baseline CA. We observed relatively constant aging rates and consistent sex differences across all BAs. Importantly, smoking, physical activity, and alcohol consumption were consistently associated with both BA and age acceleration (AgeAccel) measures. Furthermore, all five BA slopes - representing the average rate of biological aging - were associated with all-cause mortality, with the strongest associations observed for PCPhenoAge.

### DNAm clocks and aging

Epigenetic clocks have emerged as a valuable tool for assessing biological age in cross-sectional settings [2]. Our results extend these findings and support the applicability and use of epigenetics clocks in longitudinal contexts. The strong correlation between BA and CA supports the utility of age-calibrated BA measures in tracking individual aging status. These features suggest that BAs could serve as practical and interpretable biomarkers of age-related diseases in geriatric and public health settings. The strong correlation between BAs and CA largely explains the linear patterns observed in BA trajectories. Li, X. et al. reported a nonlinear association between BA and CA [31]. Nevertheless, when comparing our mixed-effect linear model with the mixed spline model employed by Li, X. et al., we did not observe any dramatic differences in model performance (data not shown).

Although all clocks in our study were blood-based, their biological targets differ: Horvath and SkinBloodClock are multi-tissue clocks, Hannum is blood-specific, and PhenoAge and GrimAge are designed as lifespan and health span predictors [8, 19–22]. As expected, AgeAccel measures - defined as residuals from regressing BA on CA - were uncorrelated with CA at both time points. In our study, the correlation patterns and regression coefficients of AgeAccel mirrored those of BA, due to their direct mathematical relationship.

### Sex difference in DNAm aging

Consistent with previous research [32–34], we observed significant sex differences in BA and AgeAccel, with women exhibiting consistently lower values than men (PCHorvath, PCHannum and PCGrimAge). These findings are in line with well-established demographic patterns showing that women generally experience longer life expectancy and delayed biological decline compared with men, despite a higher burden of non-fatal morbidity [32, 35]. Notably, while BA levels differed by sex, the overall shape of the longitudinal BA trajectories did not, suggesting that biological aging may progress at a broadly similar rate in older men and women. This observation is consistent with longitudinal twin studies reporting comparable DNA methylation aging rates across sexes in later life [36]. Together, these findings suggest that sex differences in biological aging among older adults may be primarily reflected in level differences established earlier in life, rather than in divergent aging rates during older age. Previous research has proposed that sex-related epigenetic differences may originate during early developmental or reproductive periods and attenuate later in life [37], Our results partially support this framework by indicating stable aging rates across sexes, while also suggesting that substantial sex differences in biological age levels persist into later life. These persistent differences may reflect long-term cumulative effects of sex-specific hormonal regulation, immune function, and differential lifetime exposures, rather than ongoing divergence in aging pace. Taken together, our findings underscore the importance of sex-stratified analyses in epigenetic aging research and highlight that biological age levels and aging rates may capture complementary aspects of sex differences in aging. Such distinctions are relevant for the development of targeted interventions and population-level strategies aimed at promoting healthy aging.

### Lifestyle factors and DNAm clocks

Lifestyle factors—particularly smoking, physical activity, alcohol consumption, and BMI—were significantly associated with all five BAs. Smoking is a well-known modifier of DNAm and is captured in PCGrimAge through surrogates such as DNAm-based pack-years [22, 38]. In our study, smoking was most strongly associated with PCGrimAge, especially among men. Notably, the long-term biological impact of tobacco exposure appears to persist even among former smokers, potentially contributing to accelerated aging [39]. We found significant associations of BMI with PCPhenoAge and PCGrimAge and corresponding AgeAccels only among women at two waves. While the bidirectional relationship between obesity and DNAm alterations has been discussed, accumulating evidence suggests that changes in DNA methylation are more likely a consequence of obesity rather than a causal factor [40]. Physical activity exhibited a non-linear association with BA. Moderate and low activity levels were linked to the greatest longevity benefit, while very high activity did not confer additional advantages. Individuals with either very low or very high activity levels exhibited comparable BA levels, consistent with recent findings [41, 42]. In contrast to smoking, alcohol consumption showed inverse associations with several BA measures and aging slopes in men. However, these findings should be interpreted with caution and should not be construed as evidence for a protective effect of alcohol on biological aging. The relationship between alcohol consumption and biological aging remains inconclusive. A previous study found both low and heavy alcohol consumption to be associated with accelerated biological aging, while moderate consumption was linked to decelerated aging [43]. More recent evidence suggested alcohol intake to be consistently positively associated with GrimAgeAccel, whereas for other clocks variable, often negative, associations were observed [44–46]. Consistent with these findings, our study also did not observe any significant association between alcohol consumption and BA among women [44].

### Education and DNAm clocks

Increased socioeconomic stress and disadvantage, including education, income, wealth, occupation, and childhood socioeconomic status, has been proposed as a mediator linking education and DNAm aging [47, 48]. In contrast to previous findings, our study found an inverse association of education and PCGrimAge and corresponding AgeAccel only among men with intermediate educational attainment [48–50], .

### DNAm clocks and mortality

The BAs evaluated in our study are well-established aging indicators and were found to be associated with mortality in previous studies [8, 19–22]. Our findings corroborate this evidence by showing that steeper slopes in all five BA trajectories were predictive of higher all-cause mortality, with the slope of PCPhenoAge demonstrating the strongest association. Sex specific analyses revealed that mortality associations were generally more pronounced in men. From a clinical and public health perspective, our results suggest that longitudinal changes in biological age, may be particularly informative for identifying individuals with accelerated aging trajectories who are at increased risk of adverse outcomes. If validated in additional cohorts, BA slopes could complement established risk factors by capturing cumulative biological stress over time. Although repeated DNA methylation measurements are not yet routinely implemented in clinical practice, continued methodological advances and declining costs may make longitudinal epigenetic monitoring feasible in research settings and, eventually, for risk stratification, preventive interventions and cancer survivor monitoring.

### Strengths and limitations

This study has several strengths. First, we simultaneously evaluated five PC-based DNAm clocks in the same cohort, enabling comparison across different biological aging models. Second, by leveraging repeated DNA methylation measurements, our longitudinal design enabled the assessment of individual-level trajectories and rates of biological aging rather than relying solely on biological age levels measured at a single point of time, allowing us to characterize biological aging as a dynamic process over an 8-year period and to link these aging rates to subsequent 12-year all-cause mortality outcomes. Third, comprehensive baseline data - including GP-verified clinical assessments and validated questionnaires - allowed for detailed adjustment of potential confounders. Nonetheless, several limitations should be noted. The sample was limited to ~ 900 individuals randomly selected from a single cohort of older adults from Germany, potentially limiting generalizability. Due to the longitudinal design requiring two measurements of DNA methylation 8 years apart, survival bias may have occurred, as only participants who survived to the 8-year follow-up were included. In addition, with only two measurement time points, it was not feasible to assess potential nonlinearity or short-term fluctuations in biological aging trajectories. Finally, although our models adjusted for major confounders, residual confounding from unmeasured variables such as diet, occupation, or changes in lifestyle over time cannot be ruled out.

## Conclusions

Despite its limitations, our study highlights the utility of DNAm-based biological clocks in capturing inter-individual differences in aging and mortality risk. While all five clocks were strongly correlated with CA, they varied in sensitivity to lifestyle factors and mortality. Smoking, physical activity, and alcohol consumption emerged as primary determinants of BA trajectories. Importantly, our findings underscore the relevance of sex-specific biological aging patterns and of the role of lifestyle factors in determining biological aging. Our results further indicate that biological aging trajectories may provide complementary information for population-level risk stratification, pending further validation and advances in the feasibility of repeated epigenetic measurements. Future studies should extend and replicate these findings in diverse populations, including younger populations, further clarify the mechanisms underlying BA-CA relationships, investigate interactions among multiple epigenetic age-related modifications to explore the potential of clinical and public health interventions to prevent accelerated biological aging and its adverse consequences.

## Supplementary Information

Below is the link to the electronic supplementary material.

Supplementary Material 1

## Data Availability

The data that support the findings of this study are not openly available due to reasons of sensitivity.

## Abbreviations

BA: Biological age
CA: Chronological age
DNAm: DNA methylation
AgeAccel: DNA methylation age acceleration
GP: General practitioner
BMI: Body mass index
PC: Principal component

## Declarations

### Abbreviations

Biological age.

CA Chronological age.

DNAm DNA methylation.

AgeAccel DNA methylation age acceleration.

GP General practitioner.

BMI Body mass index.

PC Principal component.
