# Supplementary material for: Tracking DNA methylation-based biological age over 8 years and its association with mortality in community-dwelling older adults
Source: Clin Epigenetics. 2026 Apr 16;18:65. doi: 10.1186/s13148-026-02067-3 (PMC13085702; doi:10.1186/s13148-026-02067-3)
Supplement: Supplementary file 1 — Supplementary Material 1 [file 13148_2026_2067_MOESM1_ESM.docx]

**Tracking DNA methylation based biological age:**

**longitudinal analyses in a cohort of community-dwelling older adults**

Qiming Yin^a,b^, Ben Schöttker^a,c^, Bernd Holleczek^d^, Ziwen Fan^a^, Joshua Stevenson-Hoare^a^, Hermann Brenner ^c⁎^

a Division of Clinical Epidemiology and Aging Research, German Cancer Research Center (DKFZ), Im Neuenheimer Feld 581, 69120 Heidelberg, Germany

b Medical Faculty Heidelberg, Heidelberg University, Im Neuenheimer Feld 672, 69120 Heidelberg, Germany

c Network Aging Research, Heidelberg University, Bergheimer Straße 20, 69115 Heidelberg, Germany

d Saarland Cancer Registry, Neugeländstraße 9, 66119 Saarbrücken, Germany

Corresponding author:

Prof. Hermann Brenner,

German Cancer Research Center (DKFZ),

Im Neuenheimer Feld 280

69120 Heidelberg, Germany.

Email: h.brenner@dkfz.de

**Table S1**. Associations of baseline characteristics with baseline PC-clocks among male participants.

| Variables | PCHorvath | | PCSkinBloodClock | | PCHannum | | PCPhenoAge | | PCGrimAge | |
| --- | --- | --- | --- | --- | --- | --- | --- | --- | --- | --- |
|  | β (95%CI) | *p*-value | β (95%CI) | *p*-value | β (95%CI) | *p*-value | β (95%CI) | *p*-value | β (95%CI) | *p*-value |
| Education ^a^ |  |  |  |  |  |  |  |  |  |  |
| Low (≤ 9 years) | ref | - | ref | - | ref | - | ref | - | ref | - |
| Intermediate | -0.77 (-1.60 - 0.07) | 0.071 | -0.80 (-1.65 - 0.05) | 0.067 | -0.83 (-1.62 - -0.03) | 0.042 | -0.87 (-1.75 - 0) | 0.050 | -0.95 (-1.47 - -0.42) | **1.79E-4** |
| High (≥ 12 years) | -0.11 (-1.14 - 0.92) | 0.834 | 0.10 (-0.95 - 1.16) | 0.847 | -0.14 (-1.12 - 0.85) | 0.783 | 0.39 (-0.69 - 1.47) | 0.479 | -0.28 (-0.93 - 0.38) | 0.409 |
| BMI |  |  |  |  |  |  |  |  |  |  |
| Underweight & Normal range (< 25) | ref | - | ref | - | ref | - | ref | - | ref | - |
| Overweight (< 30) | -0.17 (-1.08 - 0.74) | 0.711 | -0.05 (-0.98 - 0.88) | 0.919 | -0.05 (-0.92 - 0.81) | 0.904 | 0.38 (-0.57 - 1.34) | 0.428 | 0.41 (-0.17 - 0.98) | 0.164 |
| Obese (≥ 30) | 0.21 (-0.52 - 0.94) | 0.569 | 0.55 (-0.19 - 1.30) | 0.146 | 0.40 (-0.30 - 1.10) | 0.263 | 0.48 (-0.28 - 1.25) | 0.215 | -0.08 (-0.54 - 0.38) | 0.741 |
| Smoking status ^b^ |  |  |  |  |  |  |  |  |  |  |
| Never smoker | ref | - | ref | - | ref | - | ref | - | ref | - |
| Former smoker | 0.27 (-0.74 - 1.27) | 0.601 | 0.36 (-0.67 - 1.38) | 0.493 | 0.65 (-0.31 - 1.60) | 0.183 | 0.75 (-0.30 - 1.80) | 0.161 | 1.72 (1.08 - 2.35) | **1.80E-7** |
| Current smoker | 1.52 (0.12 - 2.93) | 0.034 | 1.95 (0.51 - 3.39) | **0.008** | 2.14 (0.80 - 3.48) | **0.002** | 2.89 (1.41 - 4.36) | **1.39E-4** | 5.97 (5.08 - 6.86) | **1.13E-32** |
| Physical activities ^c^ |  |  |  |  |  |  |  |  |  |  |
| Inactive | ref | - | ref | - | ref | - | ref | - | ref | - |
| Low | -0.53 (-1.77 - 0.71) | 0.403 | -0.22 (-1.49 - 1.05) | 0.737 | -0.16 (-1.35 - 1.02) | 0.786 | -0.62 (-1.92 - 0.68) | 0.346 | -0.30 (-1.09 - 0.48) | 0.446 |
| Medium or high | 0.58 (-0.31 - 1.46) | 0.200 | 0.24 (-0.67 - 1.14) | 0.609 | 0.38 (-0.46 - 1.23) | 0.371 | 0.56 (-0.36 - 1.48) | 0.235 | 0.31 (-0.24 - 0.87) | 0.270 |
| Alcohol consumption **^d^** |  |  |  |  |  |  |  |  |  |  |
| Abstainer | ref | - | ref | - | ref | - | ref | - | ref | - |
| Low | -0.22 (-1.71 - 1.27) | 0.773 | -0.36 (-1.89 - 1.16) | 0.639 | -0.12 (-1.54 - 1.31) | 0.872 | 0.27 (-1.30 - 1.83) | 0.737 | 0.06 (-0.88 - 1.00) | 0.901 |
| Medium or High | -0.16 (-1.11 - 0.78) | 0.735 | -0.35 (-1.32 - 0.62) | 0.479 | -0.16 (-1.06 - 0.75) | 0.734 | 0.50 (-0.49 - 1.49) | 0.323 | 0.14 (-0.46 - 0.74) | 0.640 |
| β and *p*-value were estimated by multivariate regression adjusted for covariates.  The bold *p*-value means passed Bonferroni-correction.  a Data missing for 11 participants without education data  b Data missing for 8 participants.  c Data missing for 2 participants. d Data missing for 19 participants. The consumption of alcohol was calculated by the following equation: 1 bottle of beer = 11.88 g ethanol, 1 glass of wine = 22.0 g ethanol, 1 shot of liquor = 6.4 g ethanol. Abstainer was without any alcohol consumption. Women 0-19.99 g/day or men 0-39.99 g/day were low consumption, and women ≥ 20 g/day or men ≥ 40 g/day were medium or high consumption. | | | | | | | | | | |

**Table S2**. Associations of baseline characteristics with baseline PC-clocks among female participants.

| Variables | PCHorvath | | PCSkinBloodClock | | PCHannum | | PCPhenoAge | | PCGrimAge | |
| --- | --- | --- | --- | --- | --- | --- | --- | --- | --- | --- |
|  | β (95%CI) | *p*-value | β (95%CI) | *p*-value | β (95%CI) | *p*-value | β (95%CI) | *p*-value | β (95%CI) | *p*-value |
| Education ^a^ |  |  |  |  |  |  |  |  |  |  |
| Low (≤ 9 years) | ref | - | ref | - | ref | - | ref | - | ref | - |
| Intermediate | -0.09 (-0.97 - 0.79) | 0.840 | 0.23 (-0.66 - 1.13) | 0.611 | -0.24 (-1.10 - 0.62) | 0.579 | 0.09 (-0.85 - 1.02) | 0.855 | 0.04 (-0.45 - 0.54) | 0.866 |
| High (≥ 12 years) | -0.25 (-1.12 - 0.62) | 0.571 | 0.06 (-0.83 - 0.95) | 0.890 | -0.18 (-1.03 - 0.67) | 0.681 | 0.27 (-0.66 - 1.19) | 0.573 | 0.02 (-0.47 - 0.51) | 0.933 |
| BMI |  |  |  |  |  |  |  |  |  |  |
| Underweight & Normal range (< 25) | ref | - | ref | - | ref | - | ref | - | ref | - |
| Overweight (< 30) | 0.55 (-0.15 - 1.25) | 0.124 | 0.41 (-0.31 - 1.13) | 0.262 | 0.54 (-0.15 - 1.22) | 0.127 | 1.30 (0.55 - 2.05) | **7.17E-4** | 0.60 (0.20 - 1.00) | **0.003** |
| Obese (≥ 30) | 0.75 (0.16 - 1.34) | 0.013 | 0.44 (-0.16 - 1.04) | 0.153 | 0.42 (-0.15 - 1.00) | 0.150 | 0.56 (-0.06 - 1.19) | 0.079 | 0.16 (-0.18 - 0.49) | 0.354 |
| Smoking status ^b^ |  |  |  |  |  |  |  |  |  |  |
| Never smoker | ref | - | ref | - | ref | - | ref | - | ref | - |
| Former smoker | -0.30 (-1.16 - 0.56) | 0.497 | -0.15 (-1.02 - 0.73) | 0.737 | -0.32 (-1.16 - 0.52) | 0.449 | 0.07 (-0.84 - 0.98) | 0.881 | 1.47 (0.99 - 1.96) | **4.87E-9** |
| Current smoker | 0.75 (-0.48 - 1.97) | 0.233 | 1.12 (-0.13 - 2.37) | 0.080 | 0.41 (-0.79 - 1.61) | 0.503 | 1.65 (0.34 - 2.96) | 0.014 | 5.59 (4.90 - 6.28) | **1.76E-44** |
| Physical activities ^c^ |  |  |  |  |  |  |  |  |  |  |
| Inactive | ref | - | ref | - | ref | - | ref | - | ref | - |
| Low | -0.70 (-1.49 - 0.08) | 0.080 | -0.97 (-1.77 - -0.16) | 0.018 | -0.91 (-1.68 - -0.14) | 0.020 | -1.32 (-2.16 - -0.48) | **0.002** | -0.58 (-1.03 - -0.14) | **0.010** |
| Medium or high | 0.21 (-0.40 - 0.82) | 0.496 | 0.57 (-0.05 - 1.19) | 0.073 | 0.15 (-0.45 - 0.74) | 0.630 | 0.45 (-0.20 - 1.09) | 0.178 | 0.16 (-0.18 - 0.50) | 0.363 |
| Alcohol consumption **^d^** |  |  |  |  |  |  |  |  |  |  |
| Abstainer | ref | - | ref | - | ref | - | ref | - | ref | - |
| Low | 0.51 (-0.52 - 1.54) | 0.333 | 0.31 (-0.75 - 1.36) | 0.567 | 0.56 (-0.45 - 1.57) | 0.276 | 0.5 (-0.6 - 1.6) | 0.369 | 0.01 (-0.57 - 0.59) | 0.971 |
| Medium or High | 0.02 (-0.67 - 0.72) | 0.946 | 0.02 (-0.69 - 0.72) | 0.964 | 0.45 (-0.22 - 1.13) | 0.189 | 0.33 (-0.41 - 1.07) | 0.376 | 0.09 (-0.3 - 0.48) | 0.662 |
| β and *p*-value were estimated by multivariate regression adjusted for covariates.  The bold *p*-value means passed Bonferroni-correction.  a Data missing for 10 participants without education data  b Data missing for 20 participants.  c Data missing for 2 participants.  d Data missing for 52 participants. The consumption of alcohol was calculated by the following equation: 1 bottle of beer = 11.88 g ethanol, 1 glass of wine = 22.0 g ethanol, 1 shot of liquor = 6.4 g ethanol. Abstainer was without any alcohol consumption. Women 0-19.99 g/day or men 0-39.99 g/day were low consumption, and women ≥ 20 g/day or men ≥ 40 g/day were medium or high consumption. | | | | | | | | | | |

**Table S3**. Associations of baseline characteristics with 8-year follow-up PC-clocks among male participants.

| Variables | PCHorvath | | PCSkinBloodClock | | PCHannum | | PCPhenoAge | | PCGrimAge | |
| --- | --- | --- | --- | --- | --- | --- | --- | --- | --- | --- |
|  | β (95%CI) | *p*-value | β (95%CI) | *p*-value | β (95%CI) | *p*-value | β (95%CI) | *p*-value | β (95%CI) | *p*-value |
| Education ^a^ |  |  |  |  |  |  |  |  |  |  |
| Low (≤ 9 years) | ref | - | ref | - | ref | - | ref | - | ref | - |
| Intermediate | -0.90 (-1.91 - 0.12) | 0.084 | -0.90 (-1.94 - 0.14) | 0.089 | -0.91 (-1.90 - 0.08) | 0.072 | -1.01 (-2.10 - 0.08) | 0.068 | -0.75 (-1.27 - -0.24) | **0.004** |
| High (≥ 12 years) | -0.81 (-2.07 - 0.44) | 0.203 | -0.43 (-1.71 - 0.85) | 0.512 | -0.74 (-1.96 - 0.48) | 0.232 | 0.09 (-1.25 - 1.43) | 0.895 | -0.39 (-1.02 - 0.25) | 0.230 |
| BMI |  |  |  |  |  |  |  |  |  |  |
| Underweight & Normal range (< 25) | ref | - | ref | - | ref | - | ref | - | ref | - |
| Overweight (< 30) | -0.39 (-1.49 - 0.71) | 0.490 | -0.35 (-1.48 - 0.77) | 0.535 | -0.17 (-1.24 - 0.90) | 0.754 | 0.32 (-0.85 - 1.50) | 0.590 | 0.46 (-0.10 - 1.01) | 0.107 |
| Obese (≥ 30) | 0.46 (-0.42 - 1.34) | 0.305 | 0.75 (-0.15 - 1.65) | 0.102 | 0.62 (-0.24 - 1.48) | 0.154 | 0.79 (-0.15 - 1.74) | 0.101 | 0.01 (-0.44 - 0.46) | 0.971 |
| Smoking status ^b^ |  |  |  |  |  |  |  |  |  |  |
| Never smoker | ref | - | ref | - | ref | - | ref | - | ref | - |
| Former smoker | 0.82 (-0.41 - 2.04) | 0.190 | 0.77 (-0.47 - 2.02) | 0.223 | 1.16 (-0.03 - 2.35) | 0.057 | 1.16 (-0.15 - 2.47) | 0.081 | 1.56 (0.94 - 2.18) | **1.08E-6** |
| Current smoker | 3.08 (1.39 - 4.76) | **3.82E-4** | 3.58 (1.86 - 5.30) | **5.29E-5** | 3.62 (1.98 - 5.26) | **1.81E-5** | 4.28 (2.47 - 6.09) | **4.41E-6** | 5.33 (4.48 - 6.18) | **3.07E-29** |
| Physical activities ^c^ |  |  |  |  |  |  |  |  |  |  |
| Inactive | ref | - | ref | - | ref | - | ref | - | ref | - |
| Low | -0.35 (-1.85 - 1.16) | 0.652 | 0.15 (-1.39 - 1.68) | 0.851 | -0.10 (-1.56 - 1.37) | 0.895 | -0.48 (-2.09 - 1.13) | 0.557 | -0.35 (-1.12 - 0.41) | 0.361 |
| Medium or high | 0.86 (-0.21 - 1.93) | 0.116 | 0.57 (-0.52 - 1.66) | 0.306 | 0.69 (-0.35 - 1.73) | 0.193 | 0.93 (-0.22 - 2.07) | 0.113 | 0.33 (-0.21 - 0.87) | 0.234 |
| Alcohol consumption **^d^** |  |  |  |  |  |  |  |  |  |  |
| Abstainer | ref | - | ref | - | ref | - | ref | - | ref | - |
| Low | -2.44 (-4.25 - -0.63) | **0.008** | -2.69 (-4.54 - -0.85) | **0.004** | -2.36 (-4.12 - -0.60) | **0.009** | -2.06 (-4.00 - -0.12) | 0.037 | -0.27 (-1.18 - 0.65) | 0.568 |
| Medium or High | -1.38 (-2.53 - -0.23) | 0.019 | -1.47 (-2.65 - -0.30) | 0.014 | -1.46 (-2.58 - -0.35) | 0.011 | -0.98 (-2.21 - 0.25) | 0.119 | -0.13 (-0.72 - 0.45) | 0.649 |
| β and *p*-value were estimated by multivariate regression adjusted for covariates.  The bold *p*-value means passed Bonferroni-correction.  a Data missing for 11 participants without education data  b Data missing for 8 participants.  c Data missing for 2 participants.  d Data missing for 19 participants. The consumption of alcohol was calculated by the following equation: 1 bottle of beer = 11.88 g ethanol, 1 glass of wine = 22.0 g ethanol, 1 shot of liquor = 6.4 g ethanol. Abstainer was without any alcohol consumption. Women 0-19.99 g/day or men 0-39.99 g/day were low consumption, and women ≥ 20 g/day or men ≥ 40 g/day were medium or high consumption. | | | | | | | | | | |

**Table S4**. Associations of baseline characteristics with 8-year follow-up PC-clocks among female participants.

| Variables | PCHorvath | | PCSkinBloodClock | | PCHannum | | PCPhenoAge | | PCGrimAge | |
| --- | --- | --- | --- | --- | --- | --- | --- | --- | --- | --- |
|  | β (95%CI) | *p*-value | β (95%CI) | *p*-value | β (95%CI) | *p*-value | β (95%CI) | *p*-value | β (95%CI) | *p*-value |
| Education ^a^ |  |  |  |  |  |  |  |  |  |  |
| Low (≤ 9 years) | ref | - | ref | - | ref | - | ref | - | ref | - |
| Intermediate | -0.28 (-1.23 - 0.67) | 0.565 | 0.03 (-0.98 - 1.05) | 0.952 | -0.42 (-1.38 - 0.54) | 0.392 | -0.39 (-1.41 - 0.63) | 0.453 | -0.09 (-0.58 - 0.39) | 0.705 |
| High (≥ 12 years) | -0.06 (-1.00 - 0.88) | 0.903 | 0.01 (-1.00 - 1.01) | 0.989 | -0.07 (-1.02 - 0.89) | 0.887 | -0.26 (-1.27 - 0.75) | 0.612 | 0.02 (-0.46 - 0.50) | 0.943 |
| BMI |  |  |  |  |  |  |  |  |  |  |
| Underweight & Normal range (< 25) | ref | - | ref | - | ref | - | ref | - | ref | - |
| Overweight (< 30) | 0.39 (-0.37 - 1.14) | 0.316 | 0.25 (-0.56 - 1.06) | 0.552 | 0.56 (-0.21 - 1.33) | 0.156 | 1.37 (0.55 - 2.18) | **0.001** | 0.63 (0.24 - 1.02) | **0.001** |
| Obese (≥ 30) | 0.89 (0.26 - 1.53) | **0.006** | 0.73 (0.05 - 1.42) | 0.036 | 0.76 (0.11 - 1.41) | 0.022 | 0.82 (0.13 - 1.51) | 0.020 | 0.31 (-0.02 - 0.64) | 0.066 |
| Smoking status ^b^ |  |  |  |  |  |  |  |  |  |  |
| Never smoker | ref | - | ref | - | ref | - | ref | - | ref | - |
| Former smoker | 0.05 (-0.88 - 0.98) | 0.914 | 0.22 (-0.78 - 1.21) | 0.668 | 0.05 (-0.89 - 0.99) | 0.917 | 0.13 (-0.87 - 1.13) | 0.803 | 1.30 (0.82 - 1.77) | **1.46E-7** |
| Current smoker | 1.55 (0.26 - 2.84) | 0.018 | 1.67 (0.29 - 3.05) | 0.018 | 1.25 (-0.06 - 2.56) | 0.062 | 2.02 (0.63 - 3.41) | **0.004** | 5.09 (4.43 - 5.75) | **3.98E-41** |
| Physical activities ^c^ |  |  |  |  |  |  |  |  |  |  |
| Inactive | ref | - | ref | - | ref | - | ref | - | ref | - |
| Low | -0.73 (-1.59 - 0.13) | 0.095 | -0.90 (-1.82 - 0.01) | 0.054 | -0.97 (-1.85 - -0.10) | 0.029 | -1.26 (-2.19 - -0.34) | **0.008** | -0.44 (-0.88 - 0) | 0.052 |
| Medium or high | 0.04 (-0.62 - 0.70) | 0.903 | 0.40 (-0.30 - 1.11) | 0.260 | 0.12 (-0.55 - 0.78) | 0.736 | 0.35 (-0.36 - 1.05) | 0.339 | 0.13 (-0.21 - 0.47) | 0.459 |
| Alcohol consumption **^d^** |  |  |  |  |  |  |  |  |  |  |
| Abstainer | ref | - | ref | - | ref | - | ref | - | ref | - |
| Low | 0.46 (-0.64 - 1.57) | 0.411 | 0.06 (-1.12 - 1.25) | 0.915 | 0.49 (-0.64 - 1.62) | 0.394 | 0.48 (-0.72 - 1.67) | 0.433 | -0.06 (-0.63 - 0.51) | 0.838 |
| Medium or High | 0.05 (-0.70 - 0.80) | 0.900 | -0.15 (-0.96 - 0.65) | 0.710 | 0.27 (-0.49 - 1.03) | 0.487 | 0.41 (-0.40 - 1.22) | 0.321 | 0.08 (-0.30 - 0.47) | 0.667 |
| β and *p*-value were estimated by multivariate regression adjusted for covariates.  The bold *p*-value means passed Bonferroni-correction.  a Data missing for 10 participants without education data  b Data missing for 20 participants.  c Data missing for 2 participants.  d Data missing for 52 participants. The consumption of alcohol was calculated by the following equation: 1 bottle of beer = 11.88 g ethanol, 1 glass of wine = 22.0 g ethanol, 1 shot of liquor = 6.4 g ethanol. Abstainer was without any alcohol consumption. Women 0-19.99 g/day or men 0-39.99 g/day were low consumption, and women ≥ 20 g/day or men ≥ 40 g/day were medium or high consumption. | | | | | | | | | | |

**Table S5.** Associations of baseline characteristics with baseline AgeAccels among all participants

| Variables | PCHorvathAgeAccel | | PCSkinBloodClockAgeAccel | | PCHannumAgeAccel | | PCPhenoAgeAccel | | PCGrimAgeAccel | |
| --- | --- | --- | --- | --- | --- | --- | --- | --- | --- | --- |
|  | β (95%CI) | *p*-value | β (95%CI) | *p*-value | β (95%CI) | *p*-value | β (95%CI) | *p*-value | β (95%CI) | *p*-value |
| Sex |  |  |  |  |  |  |  |  |  |  |
| Female | ref | - | ref | - | ref | - | ref | - | ref | - |
| Male | 1.22 (0.58 - 1.85) | **2.02E-4** | 0.51 (-0.14 - 1.16) | 0.124 | 1.39 (0.78 - 2.01) | **1.05E-5** | 0.88 (0.21 - 1.56) | **0.010** | 2.34 (1.95 - 2.72) | **7.67E-30** |
| Education |  |  |  |  |  |  |  |  |  |  |
| Low (≤ 9 years) | ref | - | ref | - | ref | - | ref | - | ref | - |
| Intermediate | -0.47 (-1.06 - 0.12) | 0.115 | -0.36 (-0.97 - 0.24) | 0.236 | -0.60 (-1.17 - -0.03) | 0.038 | -0.47 (-1.10 - 0.15) | 0.134 | -0.57 (-0.93 - -0.22) | **0.002** |
| High (≥ 12 years) | -0.27 (-0.92 - 0.39) | 0.423 | -0.04 (-0.71 - 0.63) | 0.910 | -0.23 (-0.86 - 0.40) | 0.475 | 0.21 (-0.48 - 0.90) | 0.555 | -0.26 (-0.66 - 0.14) | 0.198 |
| BMI |  |  |  |  |  |  |  |  |  |  |
| Underweight & Normal range (< 25) | ref | - | ref | - | ref | - | ref | - | ref | - |
| Overweight (< 30) | 0.14 (-0.41 - 0.69) | 0.605 | 0.18 (-0.39 - 0.74) | 0.541 | 0.29 (-0.24 - 0.82) | 0.280 | 0.84 (0.26 - 1.42) | **0.005** | 0.52 (0.19 - 0.85) | **0.002** |
| Obese (≥ 30) | 0.48 (0.02 - 0.93) | 0.040 | 0.49 (0.03 - 0.96) | 0.039 | 0.40 (-0.04 - 0.84) | 0.074 | 0.50 (0.02 - 0.98) | 0.041 | 0.05 (-0.22 - 0.33) | 0.699 |
| Smoking status |  |  |  |  |  |  |  |  |  |  |
| Never smoker | ref | - | ref | - | ref | - | ref | - | ref | - |
| Former smoker | 0.03 (-0.61 - 0.66) | 0.938 | 0.11 (-0.54 - 0.76) | 0.738 | 0.18 (-0.44 - 0.79) | 0.572 | 0.39 (-0.28 - 1.06) | 0.258 | 1.58 (1.19 - 1.97) | **3.77E-15** |
| Current smoker | 1.1 (0.19 - 2.01) | 0.018 | 1.52 (0.59 - 2.45) | **0.001** | 1.28 (0.40 - 2.16) | **0.004** | 2.28 (1.32 - 3.24) | **3.90E-6** | 5.74 (5.19 - 6.29) | **1.94E-74** |
| Physical activities |  |  |  |  |  |  |  |  |  |  |
| Inactive | ref | - | ref | - | ref | - | ref | - | ref | - |
| Low | -0.60 (-1.27 - 0.07) | 0.080 | -0.70 (-1.39 - -0.02) | 0.044 | -0.58 (-1.23 - 0.06) | 0.077 | -1.07 (-1.78 - -0.37) | **0.003** | -0.46 (-0.86 - -0.05) | 0.028 |
| Medium or high | 0.34 (-0.15 - 0.84) | 0.175 | 0.43 (-0.08 - 0.94) | 0.099 | 0.27 (-0.21 - 0.75) | 0.269 | 0.55 (0.02 - 1.07) | 0.042 | 0.23 (-0.08 - 0.53) | 0.143 |
| Alcohol consumption **^a^** |  |  |  |  |  |  |  |  |  |  |
| Abstainer | ref | - | ref | - | ref | - | ref | - | ref | - |
| Low | 0.20 (-0.66 - 1.06) | 0.646 | 0.03 (-0.84 - 0.91) | 0.939 | 0.23 (-0.60 - 1.06) | 0.586 | 0.38 (-0.52 - 1.29) | 0.409 | -0.02 (-0.54 - 0.50) | 0.945 |
| Medium or High | -0.02 (-0.59 - 0.54) | 0.933 | -0.10 (-0.68 - 0.48) | 0.733 | 0.21 (-0.34 - 0.75) | 0.458 | 0.47 (-0.12 - 1.06) | 0.121 | 0.14 (-0.20 - 0.49) | 0.410 |
| β and *p*-value were estimated by multivariate regression adjusted for covariates.  The bold *p*-value means pass Bonferroni-correction.  a Data missing for 71 participants. The consumption of alcohol was calculated by the following equation: 1 bottle of beer = 11.88 g ethanol, 1 glass of wine = 22.0 g ethanol, 1 shot of liquor = 6.4 g ethanol. Abstainer was without any alcohol consumption. Women 0-19.99 g/day or men 0-39.99 g/day were low consumption, and women ≥ 20 g/day or men ≥ 40 g/day were medium or high consumption. | | | | | | | | | | |

**Table S6.** Associations of baseline characteristics with 8-year follow-up AgeAccels among all participants

| Variables | PCHorvathAgeAccel | | PCSkinBloodClockAgeAccel | | PCHannumAgeAccel | | PCPhenoAgeAccel | | PCGrimAgeAccel | |
| --- | --- | --- | --- | --- | --- | --- | --- | --- | --- | --- |
|  | β (95%CI) | *p*-value | β (95%CI) | *p*-value | β (95%CI) | *p*-value | β (95%CI) | *p*-value | β (95%CI) | *p*-value |
| Sex |  |  |  |  |  |  |  |  |  |  |
| Female | ref | - | ref | - | ref | - | ref | - | ref | - |
| Male | 0.99 (0.24 - 1.75) | **0.010** | 0.18 (-0.60 - 0.96) | 0.653 | 1.14 (0.39 - 1.89) | **0.003** | 0.61 (-0.20 - 1.42) | 0.142 | 2.22 (1.83 - 2.60) | **6.79E-28** |
| Education |  |  |  |  |  |  |  |  |  |  |
| Low (≤ 9 years) | ref | - | ref | - | ref | - | ref | - | ref | - |
| Intermediate | -0.65 (-1.34 - 0.03) | 0.063 | -0.53 (-1.24 - 0.19) | 0.148 | -0.73 (-1.41 - -0.04) | 0.037 | -0.72 (-1.46 - 0.02) | 0.056 | -0.50 (-0.85 - -0.15) | **0.005** |
| High (≥ 12 years) | -0.53 (-1.29 - 0.24) | 0.175 | -0.35 (-1.15 - 0.44) | 0.381 | -0.53 (-1.29 - 0.23) | 0.172 | -0.21 (-1.03 - 0.62) | 0.621 | -0.25 (-0.64 - 0.14) | 0.204 |
| BMI |  |  |  |  |  |  |  |  |  |  |
| Underweight & Normal range (< 25) | ref | - | ref | - | ref | - | ref | - | ref | - |
| Overweight (< 30) | -0.03 (-0.67 - 0.61) | 0.934 | -0.06 (-0.72 - 0.61) | 0.865 | 0.20 (-0.43 - 0.84) | 0.528 | 0.82 (0.13 - 1.51) | 0.021 | 0.55 (0.23 - 0.88) | **8.89E-4** |
| Obese (≥ 30) | 0.59 (0.06 - 1.12) | 0.029 | 0.66 (0.11 - 1.21) | 0.019 | 0.61 (0.08 - 1.14) | 0.023 | 0.68 (0.10 - 1.25) | 0.021 | 0.13 (-0.14 - 0.40) | 0.349 |
| Smoking status |  |  |  |  |  |  |  |  |  |  |
| Never smoker | ref | - | ref | - | ref | - | ref | - | ref | - |
| Former smoker | 0.47 (-0.27 - 1.22) | 0.210 | 0.51 (-0.26 - 1.28) | 0.192 | 0.57 (-0.17 - 1.31) | 0.128 | 0.66 (-0.14 - 1.46) | 0.104 | 1.44 (1.06 - 1.81) | **1.64E-13** |
| Current smoker | 2.36 (1.32 - 3.41) | **9.91E-6** | 2.69 (1.61 - 3.78) | **1.26E-6** | 2.47 (1.43 - 3.50) | **3.38E-6** | 3.21 (2.09 - 4.34) | **2.83E-8** | 5.21 (4.69 - 5.74) | **1.69E-68** |
| Physical activities |  |  |  |  |  |  |  |  |  |  |
| Inactive | ref | - | ref | - | ref | - | ref | - | ref | - |
| Low | -0.55 (-1.33 - 0.23) | 0.169 | -0.54 (-1.36 - 0.27) | 0.188 | -0.65 (-1.43 - 0.12) | 0.099 | -1.00 (-1.84 - -0.16) | 0.020 | -0.39 (-0.79 - 0) | 0.051 |
| Medium or high | 0.43 (-0.15 - 1.01) | 0.145 | 0.53 (-0.07 - 1.14) | 0.083 | 0.45 (-0.13 - 1.02) | 0.129 | 0.65 (0.02 - 1.27) | 0.042 | 0.23 (-0.07 - 0.52) | 0.134 |
| Alcohol consumption **^a^** |  |  |  |  |  |  |  |  |  |  |
| Abstainer | ref | - | ref | - | ref | - | ref | - | ref | - |
| Low | -0.82 (-1.81 - 0.18) | 0.107 | -1.09 (-2.13 - -0.06) | 0.038 | -0.79 (-1.78 - 0.20) | 0.117 | -0.61 (-1.68 - 0.47) | 0.267 | -0.18 (-0.69 - 0.32) | 0.474 |
| Medium or High | -0.57 (-1.23 - 0.08) | 0.087 | -0.73 (-1.41 - -0.05) | 0.036 | -0.47 (-1.12 - 0.18) | 0.156 | -0.16 (-0.87 - 0.55) | 0.658 | 0.02 (-0.32 - 0.35) | 0.929 |
| β and *p*-value were estimated by multivariate regression adjusted for covariates.  The bold *p*-value means pass Bonferroni-correction.  a Data missing for 71 participants. The consumption of alcohol was calculated by the following equation: 1 bottle of beer = 11.88 g ethanol, 1 glass of wine = 22.0 g ethanol, 1 shot of liquor = 6.4 g ethanol. Abstainer was without any alcohol consumption. Women 0-19.99 g/day or men 0-39.99 g/day were low consumption, and women ≥ 20 g/day or men ≥ 40 g/day were medium or high consumption. | | | | | | | | | | |

**Table S7**. Associations of baseline characteristics with baseline AgeAccel among male participants.

| Variables | PCHorvathAgeAccel | | PCSkinBloodClockAgeAccel | | PCHannumAgeAccel | | PCPhenoAgeAccel | | PCGrimAgeAccel | |
| --- | --- | --- | --- | --- | --- | --- | --- | --- | --- | --- |
|  | β (95%CI) | *p*-value | β (95%CI) | *p*-value | β (95%CI) | *p*-value | β (95%CI) | *p*-value | β (95%CI) | *p*-value |
| Education ^a^ |  |  |  |  |  |  |  |  |  |  |
| Low (≤ 9 years) | ref | - | ref | - | ref | - | ref | - | ref | - |
| Intermediate | -0.77 (-1.60 - 0.07) | 0.071 | -0.80 (-1.65 - 0.05) | 0.067 | -0.83 (-1.62 - -0.03) | 0.042 | -0.87 (-1.75 - 0) | 0.050 | -0.95 (-1.47 - -0.42) | **1.79E-4** |
| High (≥ 12 years) | -0.11 (-1.14 - 0.92) | 0.834 | 0.10 (-0.95 - 1.16) | 0.847 | -0.14 (-1.12 - 0.85) | 0.783 | 0.39 (-0.69 - 1.47) | 0.479 | -0.28 (-0.93 - 0.38) | 0.409 |
| BMI |  |  |  |  |  |  |  |  |  |  |
| Underweight & Normal range (< 25) | ref | - | ref | - | ref | - | ref | - | ref | - |
| Overweight (< 30) | -0.17 (-1.08 - 0.74) | 0.711 | -0.05 (-0.98 - 0.88) | 0.919 | -0.05 (-0.92 - 0.81) | 0.904 | 0.38 (-0.57 - 1.34) | 0.428 | 0.41 (-0.17 - 0.98) | 0.164 |
| Obese (≥ 30) | 0.21 (-0.52 - 0.94) | 0.569 | 0.55 (-0.19 - 1.30) | 0.146 | 0.40 (-0.30 - 1.10) | 0.263 | 0.48 (-0.28 - 1.25) | 0.215 | -0.08 (-0.54 - 0.38) | 0.741 |
| Smoking status ^b^ |  |  |  |  |  |  |  |  |  |  |
| Never smoker | ref | - | ref | - | ref | - | ref | - | ref | - |
| Former smoker | 0.27 (-0.74 - 1.27) | 0.601 | 0.36 (-0.67 - 1.38) | 0.493 | 0.65 (-0.31 - 1.60) | 0.183 | 0.75 (-0.30 - 1.80) | 0.161 | 1.72 (1.08 - 2.35) | **1.80E-7** |
| Current smoker | 1.52 (0.12 - 2.93) | 0.034 | 1.95 (0.51 - 3.39) | **0.008** | 2.14 (0.80 - 3.48) | **0.002** | 2.89 (1.41 - 4.36) | **1.39E-4** | 5.97 (5.08 - 6.86) | **1.13E-32** |
| Physical activities ^c^ |  |  |  |  |  |  |  |  |  |  |
| Inactive | ref | - | ref | - | ref | - | ref | - | ref | - |
| Low | -0.53 (-1.77 - 0.71) | 0.403 | -0.22 (-1.49 - 1.05) | 0.737 | -0.16 (-1.35 - 1.02) | 0.786 | -0.62 (-1.92 - 0.68) | 0.346 | -0.30 (-1.09 - 0.48) | 0.446 |
| Medium or high | 0.58 (-0.31 - 1.46) | 0.200 | 0.24 (-0.67 - 1.14) | 0.609 | 0.38 (-0.46 - 1.23) | 0.371 | 0.56 (-0.36 - 1.48) | 0.235 | 0.31 (-0.24 - 0.87) | 0.270 |
| Alcohol consumption **^d^** |  |  |  |  |  |  |  |  |  |  |
| Abstainer | ref | - | ref | - | ref | - | ref | - | ref | - |
| Low | -0.22 (-1.71 - 1.27) | 0.773 | -0.36 (-1.89 - 1.16) | 0.639 | -0.12 (-1.54 - 1.31) | 0.872 | 0.27 (-1.30 - 1.83) | 0.737 | 0.06 (-0.88 - 1.00) | 0.901 |
| Medium or High | -0.16 (-1.11 - 0.78) | 0.735 | -0.35 (-1.32 - 0.62) | 0.479 | -0.16 (-1.06 - 0.75) | 0.734 | 0.50 (-0.49 - 1.49) | 0.323 | 0.14 (-0.46 - 0.74) | 0.640 |
| β and *p*-value were estimated by multivariate regression adjusted for covariates.  The bold *p*-value means passed Bonferroni-correction.  a Data missing for 11 participants without education data  b Data missing for 8 participants.  c Data missing for 2 participants.  d Data missing for 19 participants. | | | | | | | | | | |

**Table S8**. Associations of baseline characteristics with baseline AgeAccel among female participants.

| Variables | PCHorvathAgeAccel | | PCSkinBloodClockAgeAccel | | PCHannumAgeAccel | | PCPhenoAgeAccel | | PCGrimAgeAccel | |
| --- | --- | --- | --- | --- | --- | --- | --- | --- | --- | --- |
|  | β (95%CI) | *p*-value | β (95%CI) | *p*-value | β (95%CI) | *p*-value | β (95%CI) | *p*-value | β (95%CI) | *p*-value |
| Education ^a^ |  |  |  |  |  |  |  |  |  |  |
| Low (≤ 9 years) | ref | - | ref | - | ref | - | ref | - | ref | - |
| Intermediate | -0.09 (-0.97 - 0.79) | 0.840 | 0.23 (-0.66 - 1.13) | 0.611 | -0.24 (-1.10 - 0.62) | 0.579 | 0.09 (-0.85 - 1.02) | 0.855 | 0.04 (-0.45 - 0.54) | 0.866 |
| High (≥ 12 years) | -0.25 (-1.12 - 0.62) | 0.571 | 0.06 (-0.83 - 0.95) | 0.890 | -0.18 (-1.03 - 0.67) | 0.681 | 0.27 (-0.66 - 1.19) | 0.573 | 0.02 (-0.47 - 0.51) | 0.933 |
| BMI |  |  |  |  |  |  |  |  |  |  |
| Underweight & Normal range (< 25) | ref | - | ref | - | ref | - | ref | - | ref | - |
| Overweight (< 30) | 0.55 (-0.15 - 1.25) | 0.124 | 0.41 (-0.31 - 1.13) | 0.262 | 0.54 (-0.15 - 1.22) | 0.127 | 1.30 (0.55 - 2.05) | **7.17E-4** | 0.60 (0.20 - 1.00) | **0.003** |
| Obese (≥ 30) | 0.75 (0.16 - 1.34) | 0.013 | 0.44 (-0.16 - 1.04) | 0.153 | 0.42 (-0.15 - 1.00) | 0.150 | 0.56 (-0.06 - 1.19) | 0.079 | 0.16 (-0.18 - 0.49) | 0.354 |
| Smoking status ^b^ |  |  |  |  |  |  |  |  |  |  |
| Never smoker | ref | - | ref | - | ref | - | ref | - | ref | - |
| Former smoker | -0.30 (-1.16 - 0.56) | 0.497 | -0.15 (-1.02 - 0.73) | 0.737 | -0.32 (-1.16 - 0.52) | 0.449 | 0.07 (-0.84 - 0.98) | 0.881 | 1.47 (0.99 - 1.96) | **4.87E-9** |
| Current smoker | 0.75 (-0.48 - 1.97) | 0.233 | 1.12 (-0.13 - 2.37) | 0.080 | 0.41 (-0.79 - 1.61) | 0.503 | 1.65 (0.34 - 2.96) | 0.014 | 5.59 (4.90 - 6.28) | **1.76E-44** |
| Physical activities ^c^ |  |  |  |  |  |  |  |  |  |  |
| Inactive | ref | - | ref | - | ref | - | ref | - | ref | - |
| Low | -0.70 (-1.49 - 0.08) | 0.080 | -0.97 (-1.77 - -0.16) | 0.018 | -0.91 (-1.68 - -0.14) | 0.020 | -1.32 (-2.16 - -0.48) | **0.002** | -0.58 (-1.03 - -0.14) | **0.010** |
| Medium or high | 0.21 (-0.40 - 0.82) | 0.496 | 0.57 (-0.05 - 1.19) | 0.073 | 0.15 (-0.45 - 0.74) | 0.630 | 0.45 (-0.20 - 1.09) | 0.178 | 0.16 (-0.18 - 0.50) | 0.363 |
| Alcohol consumption **^d^** |  |  |  |  |  |  |  |  |  |  |
| Abstainer | ref | - | ref | - | ref | - | ref | - | ref | - |
| Low | 0.51 (-0.52 - 1.54) | 0.333 | 0.31 (-0.75 - 1.36) | 0.567 | 0.56 (-0.45 - 1.57) | 0.276 | 0.5 (-0.6 - 1.6) | 0.369 | 0.01 (-0.57 - 0.59) | 0.971 |
| Medium or High | 0.02 (-0.67 - 0.72) | 0.946 | 0.02 (-0.69 - 0.72) | 0.964 | 0.45 (-0.22 - 1.13) | 0.189 | 0.33 (-0.41 - 1.07) | 0.376 | 0.09 (-0.3 - 0.48) | 0.662 |
| β and *p*-value were estimated by multivariate regression adjusted for covariates.  The bold *p*-value means passed Bonferroni-correction.  a Data missing for 10 participants without education data  b Data missing for 20 participants.  c Data missing for 2 participants.  d Data missing for 52 participants. | | | | | | | | | | |

**Table S9.** Associations of baseline characteristics with 8-year follow-up AgeAccels among male participants

| Variables | PCHorvathAgeAccel | | PCSkinBloodClockAgeAccel | | PCHannumAgeAccel | | PCPhenoAgeAccel | | PCGrimAgeAccel | |
| --- | --- | --- | --- | --- | --- | --- | --- | --- | --- | --- |
|  | β (95%CI) | *p*-value | β (95%CI) | *p*-value | β (95%CI) | *p*-value | β (95%CI) | *p*-value | β (95%CI) | *p*-value |
| Education ^a^ |  |  |  |  |  |  |  |  |  |  |
| Low (≤ 9 years) | ref | - | ref | - | ref | - | ref | - | ref | - |
| Intermediate | -0.90 (-1.91 - 0.12) | 0.084 | -0.90 (-1.94 - 0.14) | 0.089 | -0.91 (-1.90 - 0.08) | 0.072 | -1.01 (-2.10 - 0.08) | 0.068 | -0.75 (-1.27 - -0.24) | **0.004** |
| High (≥ 12 years) | -0.81 (-2.07 - 0.44) | 0.203 | -0.43 (-1.71 - 0.85) | 0.512 | -0.74 (-1.96 - 0.48) | 0.232 | 0.09 (-1.25 - 1.43) | 0.895 | -0.39 (-1.02 - 0.25) | 0.230 |
| BMI |  |  |  |  |  |  |  |  |  |  |
| Underweight & Normal range (< 25) | ref | - | ref | - | ref | - | ref | - | ref | - |
| Overweight (< 30) | -0.39 (-1.49 - 0.71) | 0.490 | -0.35 (-1.48 - 0.77) | 0.535 | -0.17 (-1.24 - 0.90) | 0.754 | 0.32 (-0.85 - 1.50) | 0.590 | 0.46 (-0.10 - 1.01) | 0.107 |
| Obese (≥ 30) | 0.46 (-0.42 - 1.34) | 0.305 | 0.75 (-0.15 - 1.65) | 0.102 | 0.62 (-0.24 - 1.48) | 0.154 | 0.79 (-0.15 - 1.74) | 0.101 | 0.01 (-0.44 - 0.46) | 0.971 |
| Smoking status ^b^ |  |  |  |  |  |  |  |  |  |  |
| Never smoker | ref | - | ref | - | ref | - | ref | - | ref | - |
| Former smoker | 0.82 (-0.41 - 2.04) | 0.190 | 0.77 (-0.47 - 2.02) | 0.223 | 1.16 (-0.03 - 2.35) | 0.057 | 1.16 (-0.15 - 2.47) | 0.081 | 1.56 (0.94 - 2.18) | **1.08E-6** |
| Current smoker | 3.08 (1.39 - 4.76) | **3.82E-4** | 3.58 (1.86 - 5.30) | **5.29E-5** | 3.62 (1.98 - 5.26) | **1.81E-5** | 4.28 (2.47 - 6.09) | **4.41E-6** | 5.33 (4.48 - 6.18) | **3.07E-29** |
| Physical activities ^c^ |  |  |  |  |  |  |  |  |  |  |
| Inactive | ref | - | ref | - | ref | - | ref | - | ref | - |
| Low | -0.35 (-1.85 - 1.16) | 0.652 | 0.15 (-1.39 - 1.68) | 0.851 | -0.10 (-1.56 - 1.37) | 0.895 | -0.48 (-2.09 - 1.13) | 0.557 | -0.35 (-1.12 - 0.41) | 0.361 |
| Medium or high | 0.86 (-0.21 - 1.93) | 0.116 | 0.57 (-0.52 - 1.66) | 0.306 | 0.69 (-0.35 - 1.73) | 0.193 | 0.93 (-0.22 - 2.07) | 0.113 | 0.33 (-0.21 - 0.87) | 0.234 |
| Alcohol consumption **^d^** |  |  |  |  |  |  |  |  |  |  |
| Abstainer | ref | - | ref | - | ref | - | ref | - | ref | - |
| Low | -2.44 (-4.25 - -0.63) | **0.008** | -2.69 (-4.54 - -0.85) | **0.004** | -2.36 (-4.12 - -0.60) | **0.009** | -2.06 (-4.00 - -0.12) | 0.037 | -0.27 (-1.18 - 0.65) | 0.568 |
| Medium or High | -1.38 (-2.53 - -0.23) | 0.019 | -1.47 (-2.65 - -0.30) | 0.014 | -1.46 (-2.58 - -0.35) | 0.011 | -0.98 (-2.21 - 0.25) | 0.119 | -0.13 (-0.72 - 0.45) | 0.649 |
| β and *p*-value were estimated by multivariate regression adjusted for covariates.  The bold *p*-value means passed Bonferroni-correction.  a Data missing for 11 participants without education data  b Data missing for 8 participants.  c Data missing for 2 participants.  d Data missing for 19 participants. | | | | | | | | | | |

**Table S10.** Associations of baseline characteristics with 8-year follow-up AgeAccels among female participants

| Variables | PCHorvathAgeAccel | | PCSkinBloodClockAgeAccel | | PCHannumAgeAccel | | PCPhenoAgeAccel | | PCGrimAgeAccel | |
| --- | --- | --- | --- | --- | --- | --- | --- | --- | --- | --- |
|  | β (95%CI) | *p*-value | β (95%CI) | *p*-value | β (95%CI) | *p*-value | β (95%CI) | *p*-value | β (95%CI) | *p*-value |
| Education ^a^ |  |  |  |  |  |  |  |  |  |  |
| Low (≤ 9 years) | ref | - | ref | - | ref | - | ref | - | ref | - |
| Intermediate | -0.28 (-1.23 - 0.67) | 0.565 | 0.03 (-0.98 - 1.05) | 0.952 | -0.42 (-1.38 - 0.54) | 0.392 | -0.39 (-1.41 - 0.63) | 0.453 | -0.09 (-0.58 - 0.39) | 0.705 |
| High (≥ 12 years) | -0.06 (-1.00 - 0.88) | 0.903 | 0.01 (-1.00 - 1.01) | 0.989 | -0.07 (-1.02 - 0.89) | 0.887 | -0.26 (-1.27 - 0.75) | 0.612 | 0.02 (-0.46 - 0.50) | 0.943 |
| BMI |  |  |  |  |  |  |  |  |  |  |
| Underweight & Normal range (< 25) | ref | - | ref | - | ref | - | ref | - | ref | - |
| Overweight (< 30) | 0.39 (-0.37 - 1.14) | 0.316 | 0.25 (-0.56 - 1.06) | 0.552 | 0.56 (-0.21 - 1.33) | 0.156 | 1.37 (0.55 - 2.18) | **0.001** | 0.63 (0.24 - 1.02) | **0.001** |
| Obese (≥ 30) | 0.89 (0.26 - 1.53) | **0.006** | 0.73 (0.05 - 1.42) | 0.036 | 0.76 (0.11 - 1.41) | 0.022 | 0.82 (0.13 - 1.51) | 0.020 | 0.31 (-0.02 - 0.64) | 0.066 |
| Smoking status ^b^ |  |  |  |  |  |  |  |  |  |  |
| Never smoker | ref | - | ref | - | ref | - | ref | - | ref | - |
| Former smoker | 0.05 (-0.88 - 0.98) | 0.914 | 0.22 (-0.78 - 1.21) | 0.668 | 0.05 (-0.89 - 0.99) | 0.917 | 0.13 (-0.87 - 1.13) | 0.803 | 1.30 (0.82 - 1.77) | **1.46E-7** |
| Current smoker | 1.55 (0.26 - 2.84) | 0.018 | 1.67 (0.29 - 3.05) | 0.018 | 1.25 (-0.06 - 2.56) | 0.062 | 2.02 (0.63 - 3.41) | **0.004** | 5.09 (4.43 - 5.75) | **3.98E-41** |
| Physical activities ^c^ |  |  |  |  |  |  |  |  |  |  |
| Inactive | ref | - | ref | - | ref | - | ref | - | ref | - |
| Low | -0.73 (-1.59 - 0.13) | 0.095 | -0.90 (-1.82 - 0.01) | 0.054 | -0.97 (-1.85 - -0.10) | 0.029 | -1.26 (-2.19 - -0.34) | **0.008** | -0.44 (-0.88 - 0) | 0.052 |
| Medium or high | 0.04 (-0.62 - 0.70) | 0.903 | 0.40 (-0.30 - 1.11) | 0.260 | 0.12 (-0.55 - 0.78) | 0.736 | 0.35 (-0.36 - 1.05) | 0.339 | 0.13 (-0.21 - 0.47) | 0.459 |
| Alcohol consumption **^d^** |  |  |  |  |  |  |  |  |  |  |
| Abstainer | ref | - | ref | - | ref | - | ref | - | ref | - |
| Low | 0.46 (-0.64 - 1.57) | 0.411 | 0.06 (-1.12 - 1.25) | 0.915 | 0.49 (-0.64 - 1.62) | 0.394 | 0.48 (-0.72 - 1.67) | 0.433 | -0.06 (-0.63 - 0.51) | 0.838 |
| Medium or High | 0.05 (-0.70 - 0.80) | 0.900 | -0.15 (-0.96 - 0.65) | 0.710 | 0.27 (-0.49 - 1.03) | 0.487 | 0.41 (-0.40 - 1.22) | 0.321 | 0.08 (-0.30 - 0.47) | 0.667 |
| β and *p*-value were estimated by multivariate regression adjusted for covariates.  The bold *p*-value means passed Bonferroni-correction  a Data missing for 10 participants without education data  b Data missing for 20 participants.  c Data missing for 2 participants.  d Data missing for 52 participants. | | | | | | | | | | |

**Table S11**. Associations of baseline characteristics with BA slopes among males.

| Variables | PCHorvath slope | | PCSkinBloodClock slope | | PCHannum slope | | PCPhenoAge slope | | PCGrimAge slope | |
| --- | --- | --- | --- | --- | --- | --- | --- | --- | --- | --- |
|  | β (95%CI) | *p*-value | β (95%CI) | *p*-value | β (95%CI) | *p*-value | β (95%CI) | *p*-value | β (95%CI) | *p*-value |
| Education ^a^ |  |  |  |  |  |  |  |  |  |  |
| Low (≤ 9 years) | ref | - | ref | - | ref | - | ref | - | ref | - |
| Intermediate | -0.04 (-0.12 - 0.04) | 0.366 | -0.04 (-0.12 - 0.04) | 0.337 | -0.04 (-0.12 - 0.05) | 0.386 | -0.02 (-0.16 - 0.11) | 0.736 | 0.03 (-0.03 - 0.08) | 0.393 |
| High (≥ 12 years) | -0.08 (-0.18 - 0.02) | 0.105 | -0.05 (-0.15 - 0.05) | 0.345 | -0.07 (-0.18 - 0.04) | 0.197 | -0.04 (-0.21 - 0.13) | 0.643 | -0.01 (-0.08 - 0.06) | 0.772 |
| BMI |  |  |  |  |  |  |  |  |  |  |
| Underweight & Normal range (< 25) | ref | - | ref | - | ref | - | ref | - | ref | - |
| Overweight (< 30) | 0 (-0.08 - 0.09) | 0.944 | 0 (-0.09 - 0.09) | 0.978 | 0.02 (-0.08 - 0.11) | 0.743 | 0.04 (-0.11 - 0.19) | 0.609 | 0.03 (-0.04 - 0.09) | 0.421 |
| Obese (≥ 30) | 0.05 (-0.02 - 0.12) | 0.165 | 0.06 (-0.02 - 0.13) | 0.129 | 0.05 (-0.02 - 0.13) | 0.177 | 0.06 (-0.06 - 0.18) | 0.332 | 0.01 (-0.04 - 0.06) | 0.672 |
| Smoking status ^b^ |  |  |  |  |  |  |  |  |  |  |
| Never smoker | ref | - | ref | - | ref | - | ref | - | ref | - |
| Former smoker | 0.05 (-0.04 - 0.15) | 0.286 | 0.05 (-0.04 - 0.15) | 0.282 | 0.05 (-0.05 - 0.16) | 0.315 | 0.07 (-0.09 - 0.24) | 0.373 | 0 (-0.07 - 0.07) | 0.937 |
| Current smoker | 0.21 (0.08 - 0.34) | **0.002** | 0.21 (0.07 - 0.35) | **0.003** | 0.22 (0.07 - 0.36) | **0.003** | 0.32 (0.09 - 0.55) | **0.006** | 0.01 (-0.09 - 0.11) | 0.826 |
| Physical activities ^c^ |  |  |  |  |  |  |  |  |  |  |
| Inactive | ref | - | ref | - | ref | - | ref | - | ref | - |
| Low | 0.06 (-0.06 - 0.18) | 0.321 | 0.08 (-0.04 - 0.21) | 0.185 | 0.05 (-0.08 - 0.17) | 0.485 | 0.05 (-0.15 - 0.25) | 0.607 | 0.02 (-0.07 - 0.10) | 0.686 |
| Medium or high | 0.04 (-0.05 - 0.12) | 0.392 | 0.02 (-0.07 - 0.11) | 0.614 | 0.04 (-0.05 - 0.13) | 0.348 | 0.09 (-0.06 - 0.23) | 0.237 | 0.03 (-0.03 - 0.09) | 0.367 |
| Alcohol consumption **^d^** |  |  |  |  |  |  |  |  |  |  |
| Abstainer | ref | - | ref | - | ref | - | ref | - | ref | - |
| Low | -0.25 (-0.40 - -0.11) | **4.58E-4** | -0.29 (-0.43 - -0.14) | **1.66E-4** | -0.29 (-0.44 - -0.13) | **3.14E-4** | -0.34 (-0.58 - -0.10) | **0.006** | -0.06 (-0.16 - 0.04) | 0.243 |
| Medium or High | -0.15 (-0.24 - -0.06) | **0.002** | -0.13 (-0.22 - -0.03) | **0.007** | -0.17 (-0.26 - -0.07) | **9.79E-4** | -0.24 (-0.39 - -0.08) | **0.003** | -0.05 (-0.11 - 0.02) | 0.151 |
| β and *p*-value were estimated by multivariate regression adjusted for covariates.  The bold *p*-value means passed Bonferroni-correction  a Data missing for 11 participants without education data  b Data missing for 8 participants.  c Data missing for 2 participants.  d Data missing for 19 participants. | | | | | | | | | | |

**Table S12**. Associations of baseline characteristics with BA slopes among females.

| Variables | Horvath’s DNAm slope | | Hannum’s DNAm slope | | PhenoAge slope | | GrimAge slope | | GrimAge2 slope | |
| --- | --- | --- | --- | --- | --- | --- | --- | --- | --- | --- |
|  | β (95%CI) | *p*-value | β (95%CI) | *p*-value | β (95%CI) | *p*-value | β (95%CI) | *p*-value | β (95%CI) | *p*-value |
| Education |  |  |  |  |  |  |  |  |  |  |
| Low (≤ 9 years) | ref | - | ref | - | ref | - | ref | - | ref | - |
| Intermediate | 0 (-0.07 - 0.07) | 0.931 | 0.01 (-0.08 - 0.10) | 0.802 | 0.01 (-0.09 - 0.10) | 0.909 | -0.03 (-0.17 - 0.12) | 0.733 | 0 (-0.06 - 0.06) | 0.964 |
| High (≥ 12 years) | 0.01 (-0.06 - 0.08) | 0.761 | 0.01 (-0.08 - 0.09) | 0.840 | -0.01 (-0.10 - 0.08) | 0.854 | -0.10 (-0.24 - 0.05) | 0.180 | -0.03 (-0.09 - 0.03) | 0.346 |
| BMI |  |  |  |  |  |  |  |  |  |  |
| Underweight & Normal range (< 25) | ref | - | ref | - | ref | - | ref | - | ref | - |
| Overweight (< 30) | 0 (-0.06 - 0.05) | 0.911 | 0 (-0.06 - 0.07) | 0.894 | 0.02 (-0.06 - 0.09) | 0.652 | 0.05 (-0.06 - 0.17) | 0.370 | 0.02 (-0.03 - 0.07) | 0.413 |
| Obese (≥ 30) | -0.01 (-0.06 - 0.04) | 0.677 | 0 (-0.06 - 0.06) | 0.930 | 0.02 (-0.05 - 0.08) | 0.625 | 0.02 (-0.08 - 0.12) | 0.709 | 0.02 (-0.02 - 0.06) | 0.337 |
| Smoking status |  |  |  |  |  |  |  |  |  |  |
| Never smoker | ref | - | ref | - | ref | - | ref | - | ref | - |
| Former smoker | 0.07 (0 - 0.14) | 0.038 | 0.10 (0.02 - 0.19) | 0.016 | 0.07 (-0.02 - 0.16) | 0.106 | 0.09 (-0.05 - 0.23) | 0.210 | 0.01 (-0.05 - 0.07) | 0.697 |
| Current smoker | 0.09 (-0.01 - 0.19) | 0.069 | 0.10 (-0.02 - 0.22) | 0.099 | 0.11 (-0.02 - 0.23) | 0.104 | 0.13 (-0.07 - 0.33) | 0.214 | -0.06 (-0.14 - 0.02) | 0.157 |
| Physical activities |  |  |  |  |  |  |  |  |  |  |
| Inactive | ref | - | ref | - | ref | - | ref | - | ref | - |
| Low | -0.05 (-0.11 - 0.02) | 0.141 | -0.07 (-0.14 - 0.01) | 0.092 | -0.08 (-0.16 - 0) | 0.064 | -0.10 (-0.23 - 0.03) | 0.125 | -0.01 (-0.06 - 0.05) | 0.800 |
| Medium or high | 0.01 (-0.04 - 0.06) | 0.789 | 0.02 (-0.04 - 0.08) | 0.603 | 0.04 (-0.02 - 0.11) | 0.195 | 0.07 (-0.03 - 0.17) | 0.172 | 0.02 (-0.02 - 0.06) | 0.282 |
| Alcohol consumption **^a^** |  |  |  |  |  |  |  |  |  |  |
| Abstainer | ref | - | ref | - | ref | - | ref | - | ref | - |
| Low | -0.02 (-0.10 - 0.06) | 0.624 | -0.04 (-0.14 - 0.07) | 0.481 | -0.04 (-0.15 - 0.07) | 0.446 | -0.09 (-0.26 - 0.08) | 0.294 | -0.03 (-0.10 - 0.04) | 0.333 |
| Medium or High | 0 (-0.05 - 0.06) | 0.926 | -0.01 (-0.08 - 0.06) | 0.748 | -0.03 (-0.10 - 0.05) | 0.495 | -0.03 (-0.14 - 0.09) | 0.620 | -0.02 (-0.06 - 0.03) | 0.529 |
| β and *p*-value were estimated by multivariate regression adjusted for covariates.  a Data missing for 10 participants without education data  b Data missing for 20 participants.  c Data missing for 2 participants.  d Data missing for 52 participants. | | | | | | | | | | |

**Table S13**. Associations of BA slopes with all-cause mortality

| Variables | model 1  (HR per SD (95%CI)) | *p*-value | model 2  (HR per SD (95%CI)) | *p*-value |
| --- | --- | --- | --- | --- |
| All participants (N = 894) | | | | |
| PCHorvath slope | 1.27 (1.13 - 1.43) | **7.08E-5** | 1.23 (1.08 - 1.40) | **0.001** |
| PCSkinBloodClock slope | 1.18 (1.05 - 1.33) | 0.006 | 1.17 (1.03 - 1.33) | 0.017 |
| PCHannum slope | 1.25 (1.10 - 1.42) | **4.76E-4** | 1.21 (1.05 - 1.39) | 0.007 |
| PCPhenoAge slope | 1.34 (1.17 - 1.53) | **1.27E-5** | 1.28 (1.11 - 1.46) | **4.28E-4** |
| PCGrimAge slope | 1.16 (1.01 - 1.32) | 0.031 | 1.18 (1.03 - 1.34) | 0.013 |
| Female (N = 486) | | | | |
| PCHorvath slope | 1.23 (1.00 - 1.51) | 0.052 | 1.22 (0.96 - 1.55) | 0.103 |
| PCSkinBloodClock slope | 1.12 (0.93 - 1.35) | 0.222 | 1.14 (0.93 - 1.39) | 0.215 |
| PCHannum slope | 1.14 (0.95 - 1.37) | 0.158 | 1.15 (0.93 - 1.41) | 0.196 |
| PCPhenoAge slope | 1.18 (0.97 - 1.43) | 0.099 | 1.21 (0.98 - 1.50) | 0.081 |
| PCGrimAge slope | 1.03 (0.84 - 1.26) | 0.764 | 1.08 (0.86 - 1.35) | 0.516 |
| Male (N = 408) | | | | |
| PCHorvath slope | 1.26 (1.09 - 1.46) | **0.001** | 1.26 (1.08 - 1.47) | 0.004 |
| PCSkinBloodClock slope | 1.21 (1.03 - 1.41) | 0.020 | 1.21 (1.02 - 1.44) | 0.028 |
| PCHannum slope | 1.35 (1.12 - 1.61) | **0.001** | 1.31 (1.07 - 1.59) | 0.008 |
| PCPhenoAge slope | 1.46 (1.23 - 1.74) | **2.12E-5** | 1.36 (1.13 - 1.64) | **0.001** |
| PCGrimAge slope | 1.27 (1.07 - 1.50) | 0.006 | 1.24 (1.05 - 1.45) | 0.009 |
| Values are Hazard Ratios (95% Confidence Interval) [HR (95%CI)] unless stated otherwise.  The bold *p*-value means passed Bonferroni-correction.  HRs (95%CIs) in each column refer to the relative risks associated with one-SD increase in the level of BA of five different models with one corresponding BA slope being the predictor of the mortality risk. Model 1 is the univariate survival model with only one BA slope taken into account. Model 2 is the multi-variate survival model, in which common risk factors (sex, education attainment, smoking status, BMI, physical activity, and alcohol consumption) were additionally adjusted for on the basis of Model 1. Attained age was used as the time-scale and thus age was inherently adjusted for. | | | | |


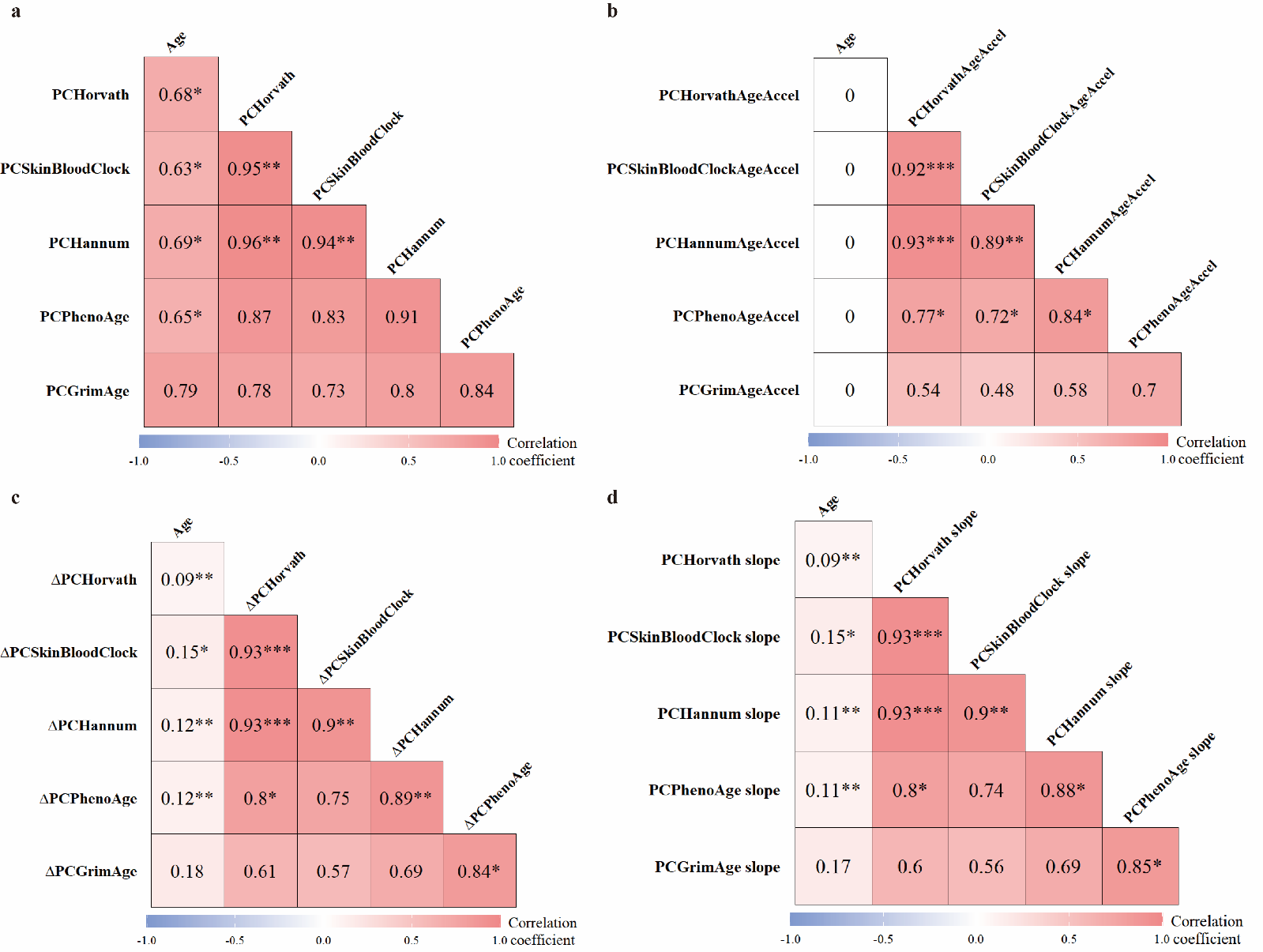
Figures

**Figure S1**. Correlation of BAs and AgeAccels. Heatmaps presenting correlation coefficients of BAs (**a**) and AgeAccels (**b**) with corresponding CA at T1. **c** and **d** present the correlation between changes of BAs (**c**), and average changing rate (slope) over interval (**d**), respectively. Red and blue tiles represented positive and negative correlations, respectively; color density indicated the magnitude of correlation coefficients. Statistical significance after Bonferroni-correction is indicated as follows: **p* < 0.05, ***p* < 0.01, ****p* < 0.001. AgeAccel, age acceleration.

**
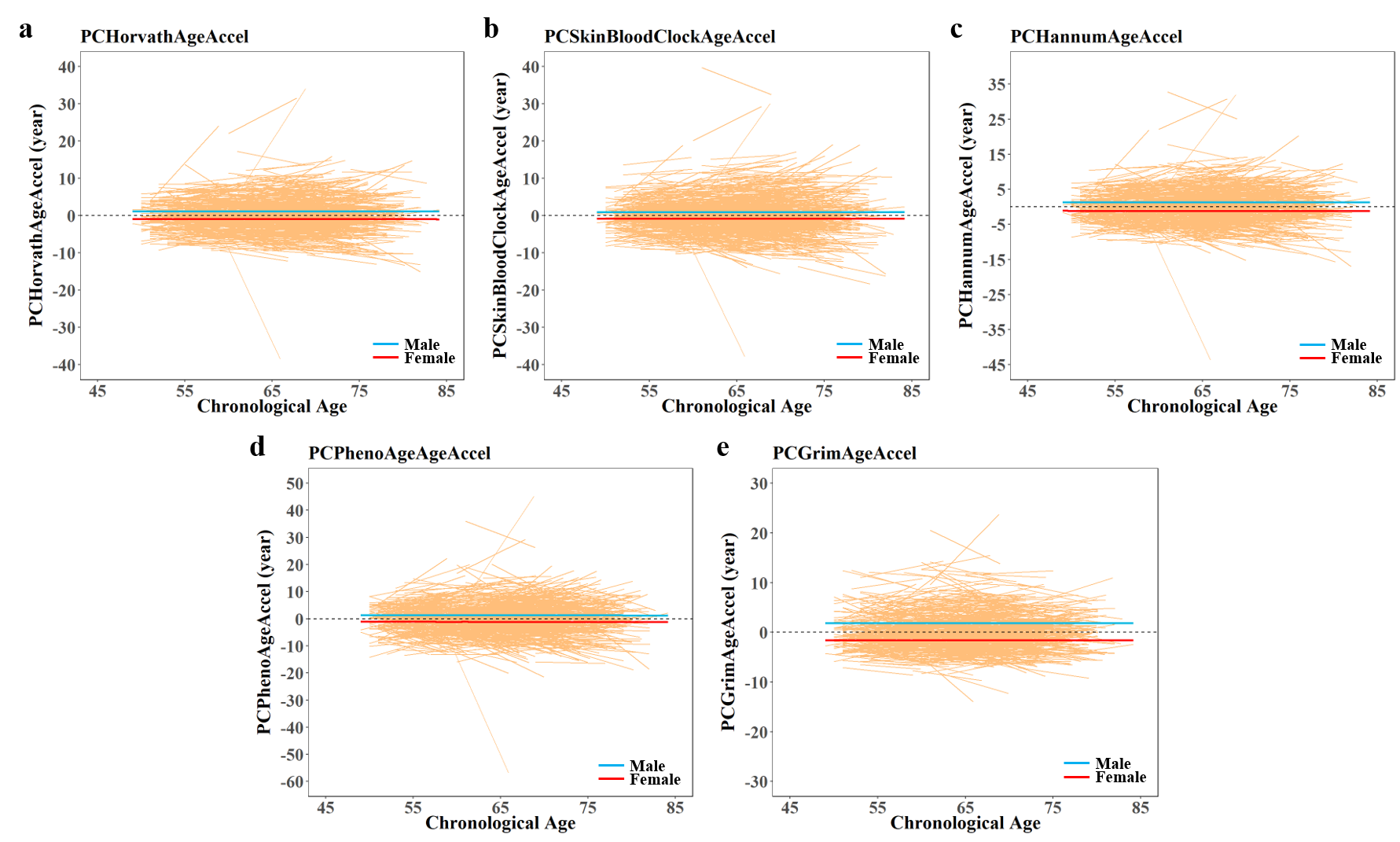
Figure S2.** Longitudinal trajectories of individual and population level AgeAccel estimates. Both individual-level AgeAccel estimates and sex-specific population AgeAccel means compared to CA are presented. Individual AgeAccel estimates are presented as orange lines as two measurements were assessed for a given individual and sex-specific population AgeAccel as blue or red smooth line. Dashed line presents slope of 0 and intercept of 0. AgeAccel, age acceleration.
